# Comparative Susceptibility of Different Populations of *Amblyomma sculptum* to *Rickettsia rickettsii*

**DOI:** 10.3389/fphys.2019.00653

**Published:** 2019-05-28

**Authors:** Monize Gerardi, Alejandro Ramírez-Hernández, Lina C. Binder, Felipe S. Krawczak, Fábio Gregori, Marcelo B. Labruna

**Affiliations:** Department of Preventive Veterinary Medicine and Animal Health, Faculty of Veterinary Medicine, University of São Paulo, São Paulo, Brazil

**Keywords:** spotted fever group *Rickettsia*, experimental infection, vector competence, transovarial transmission, transstadial maintenance

## Abstract

The bacterium *Rickettsia rickettsii* is the etiological agent of Brazilian spotted fever (BSF), which is transmitted in Brazil mainly by the tick *Amblyomma sculptum.* Herein, larvae and nymphs of six populations of *A. sculptum* were exposed to *R. rickettsii* by feeding on needle-inoculated guinea pigs, and thereafter reared on uninfected guinea pigs or rabbits. Two tick populations were exposed to autochthone *R. rickettsii* strains, whereas four tick populations were exposed to non-autochthone strains. The six geographically different populations of *A. sculptum* showed different susceptibilities to *R. rickettsii*, higher among the two tick populations that were exposed to their autochthone *R. rickettsii* strain. In addition, higher rates of transovarial transmission of *R. rickettsii* and vector competence success also included the two tick populations that were exposed to autochthone *R. rickettsii* strains. These results indicate that the susceptibility of *A. sculptum* to *R. rickettsii* varies among different tick populations, with a clear bias for higher susceptibility to an autochthone *R. rickettsii* strain that has already coevolved with a tick population for some time. Our results demonstrated that the *R. rickettsii* infection induces higher mortality of engorged larvae and nymphs, and tend to reduce the reproductive fitness of engorged females. All together, these results might explain the low *R. rickettsii-*infection rates of *A. sculptum* under natural conditions (usually <1%), and indicate that an *A. sculptum* population should not be able to sustain a *R. rickettsii* infection for successive tick generations without the creation of new cohorts of infected ticks via horizontal transmission on vertebrate rickettsemic hosts (amplifying hosts). Finally, despite of the ubiquitous distribution of *A. sculptum* in southeastern and central-western Brazil, most of the populations of this tick species are devoid of *R. rickettsii* infection. This scenario might be related to two major factors: (i) insufficient numbers of susceptible amplifying hosts; and (ii) lower susceptibilities of many tick populations. While the first factor has been demonstrated by mathematical models in previous studies, the second is highlighted by the results observed in the present study.

## Introduction

The bacterium *Rickettsia rickettsii* is the etiological agent of Brazilian spotted fever (BSF), the deadliest tick-borne disease of the New World. In southeastern Brazil, 776 cases of BSF were laboratory-confirmed in humans from 2007 to 2015, with an overall case-fatality rate of 55% (Oliveira et al., 2016). While several tick species of the genus *Amblyomma* have been implicated in the transmission of *R. rickettsii* to humans in South America (Labruna et al., 2014), *Amblyomma sculptum* (formerly called as *Amblyomma cajennense* in southeastern Brazil) is the most important vector, since most of the BSF-confirmed cases have been associated to this tick species (Pinter et al., 2011; Krawczak et al., 2014; Labruna et al., 2017).

*Amblyomma sculptum*, a member of the *A. cajennense* species complex, is by far the most frequent human-biting tick species in Brazil (Guglielmone et al., 2006; Ramos et al., 2014; Martins et al., 2016). While this may suggest a high exposure of humans to *R. rickettsii* infection, the incidence of BSF has been as low as 0.14/100,000 inhabitants (Oliveira et al., 2016), thanks to the very low *R. rickettsii-*infection rates among *A. sculptum* populations, reported to be between 0.05 and 1.28% (Guedes et al., 2005, 2011; Krawczak et al., 2014; Labruna et al., 2017). The very low *R. rickettsii-*infection rates of *A. sculptum* under natural conditions has been attributed to the lower susceptibility of this tick species to the bacterium when compared to other tick species that are found with much higher infection rates under natural conditions, such as *Amblyomma aureolatum* and *Rhipicephalus sanguineus* sensu lato (s.l.) (Labruna et al., 2008, 2011; Pacheco et al., 2011; Ogrzewalska et al., 2012; Soares et al., 2012).

*Amblyomma sculptum* has a ubiquitous distribution in southeastern and central-western Brazil (Martins et al., 2016); however, only a few populations of this tick species have been found infected by *R. rickettsii* (Sangioni et al., 2005; Pacheco et al., 2007, 2009; Soares et al., 2012). This scenario could be linked to different susceptibilities to *R. rickettsii* infection among different populations of *A. sculptum*. Previous studies that quantified the susceptibility of *A. sculptum* to *R. rickettsii* employed a rickettsial strain that was originally isolated from *A. aureolatum* ticks in an area without *A. sculptum* (Labruna et al., 2008; Soares et al., 2012). Therefore, more reliable data could be obtained with additional studies employing *A. sculptum-*derived *R. rickettsii* strains. Based on the above statements, the present study evaluated the comparative susceptibility of different populations of *A. sculptum* to the infection by *R. rickettsii*, using two rickettsial strains that were originally isolated from *A. sculptum* in Brazil.

## Materials and Methods

### Ethics Statements

This study has been approved by the Institutional Animal Care and Use Committee (IACUC) of the Faculty of Veterinary Medicine of the University of São Paulo (protocols 3104/2013 and 5948070314). Field capture of ticks was authorized by the Brazilian Ministry of the Environment (permit SISBIO Nos. 43259-1 and 11459-1).

### Ticks

A total of six geographically distinct populations of *A. sculptum* were used in this study, as detailed in Table 1. From five populations, unfed adults were collected on the vegetation by dragging or dry ice traps following Terassini et al. (2010) or Szabó et al. (2009), and brought alive to the laboratory, where adult ticks were allowed to feed on tick-naïve rabbits, in order to establish five independent tick colonies. From a sixth tick population (E-ITU), engorged females were collected from capybaras (*Hydrochoerus hydrochaeris*) and brought to the laboratory to start a tick colony.

**Table 1:** General characteristics of the local origin of the *Amblyomma sculptum* populations that originated the six tick colonies used in the present study.

Tick colony code	Origin (municipality, state)	Conservation status of the origin	Main hosts sustaining the *A. sculptum* population	Status for Brazilian spotted fever (*R. rickettsii*)	References
E-ITU	Itu, São Paulo	Highly anthropic, low biodiversity	Capybaras	Endemic	Krawczak et al., 2014
E-PIC	Piracicaba, São Paulo	Highly anthropic, low biodiversity	Capybaras	Endemic	Perez et al., 2008
E-PAM	Belo Horizonte, Minas Gerais	Highly anthropic, low biodiversity	Capybaras	Endemic	Labruna et al., 2017
NE-PIS	Pirassununga, São Paulo	Highly anthropic, low biodiversity	Capybaras	Non-endemic	Horta et al., 2007
NE-POC	Poconé, Mato Grosso	Natural area, high biodiversity	Tapirs, peccaries	Non-endemic	Melo et al., 2016
NE-GSV	Chapada Gaúcha, Minas Gerais	Natural area, high biodiversity	Tapirs, peccaries	Non-endemic	Barbieri et al., 2019

For all six tick colonies, off-host conditions were provided by an incubator set for a temperature of 27°C, 85% relative humidity and photoperiod 0:24 (light:dark). Our experimental infestations were started with the F_1_ larvae (first lab generation) of each of the six tick colonies. Before starting the experiments, we had the following evidence that these ticks were free of rickettsial infection: all engorged females that yielded the F_1_ larvae, as well as a small sample of their egg batches were negative by real-time PCR for the genus *Rickettsia* (protocol described below) at the end of oviposition; and all rabbits remained seronegative to *R. rickettsii* (protocol described below) 21 days after being infested with field-collected adult ticks.

Among the six colonies of *A. sculptum*, three were from BSF-endemic areas in which fatal cases of BSF have been reported recently, in association with the transmission by *A. sculptum*. Three other colonies were from areas where BSF was never reported (non-endemic areas), which include a highly anthropic area in the state of São Paulo, and two natural areas with a rich biodiversity (Table 1).

### *Rickettsia rickettsii* Strains

Two strains of *R. rickettsii* were used in this study, strain Itu and strain Pampulha. The former was isolated from *A. sculptum* collected in a BSF-endemic area in Itu municipality (Krawczak et al., 2014), the same area where we collected *A. sculptum* adult ticks to form our E-ITU tick colony. The later was collected in the Pampulha Lake area, a BSF-endemic area in Belo Horizonte City (Labruna et al., 2017), where we collected *A. sculptum* adult ticks to form our E-PAM tick colony. Both *R. rickettsii* strains were originally isolated through the inoculation of guinea pigs with field-collected *A. sculptum* tick homogenate and subsequently adaptation of the strain to Vero cell culture (Krawczak et al., 2014; Labruna et al., 2017). However, only the guinea pig lineage of each strain (never *in vitro* cultured) was used for experimental infections in the present study. For this purpose, each rickettsial strain has been maintained through guinea pig passages in our laboratory, and in each passage we have cryopreserved fragments of infected spleen, lung, liver, and brain. In the present study, we used fragments of these four organs of the third guinea pig passage of each isolate to inoculate guinea pigs, as described below.

### Tick Infestation Protocols

Throughout this study, larvae and nymphs were allowed to feed on tick-naïve adult male (or female in a few cases) guinea pigs, whereas adult ticks were allowed to feed on tick-naïve adult male domestic rabbits. In either case, tick infestations were performed inside cotton sleeves (10–20 cm diameter) that were glued to the shaved back of each animal, as previously described (Pinter et al., 2002; Horta et al., 2009). Ticks (larvae, nymphs or adults, 20–30 days old) were released into the sleeve. Larval infestations consisted of approximately 500–1500 larvae per guinea pig; nymphal infestations consisted of 80–100 nymphs per guinea pig; and adult infestations consisted of 7–20 couples per rabbit. The sleeves were opened daily, and the detached engorged ticks were removed, counted and immediately taken to the incubator where molting (for engorged larvae and nymphs) or oviposition (for engorged females) was observed daily. Engorged females were individually weighed in an electronic balance (precision of 0.0001 g) at the detachment day. Molting success (survivorship) was determined for engorged larvae and nymphs whereas the following biological parameters were determined for engorged females: feeding period (number of days from placement of the ticks on the host to the detachment), oviposition success (proportion of engorged females that successfully oviposited), preoviposition period (number of days from detachment to the beginning of oviposition) and egg mass incubation period (number of days from the beginning of oviposition to the hatching of the first larva). Additionally, the total egg mass laid by each female was weighed at the end of oviposition and a conversion efficiency index (CEI = mg egg mass/mg engorged female × 100), which measures the efficiency with which a tick species converts body weight into eggs (Drummond and Whetstone, 1970), was determined for each female that oviposited. Percentage of hatching for each female egg mass was visually estimated according to Labruna et al. (2000). During the experiment, animals were fed with commercial pellets and water *ad libitum*.

**FIGURE 1:**
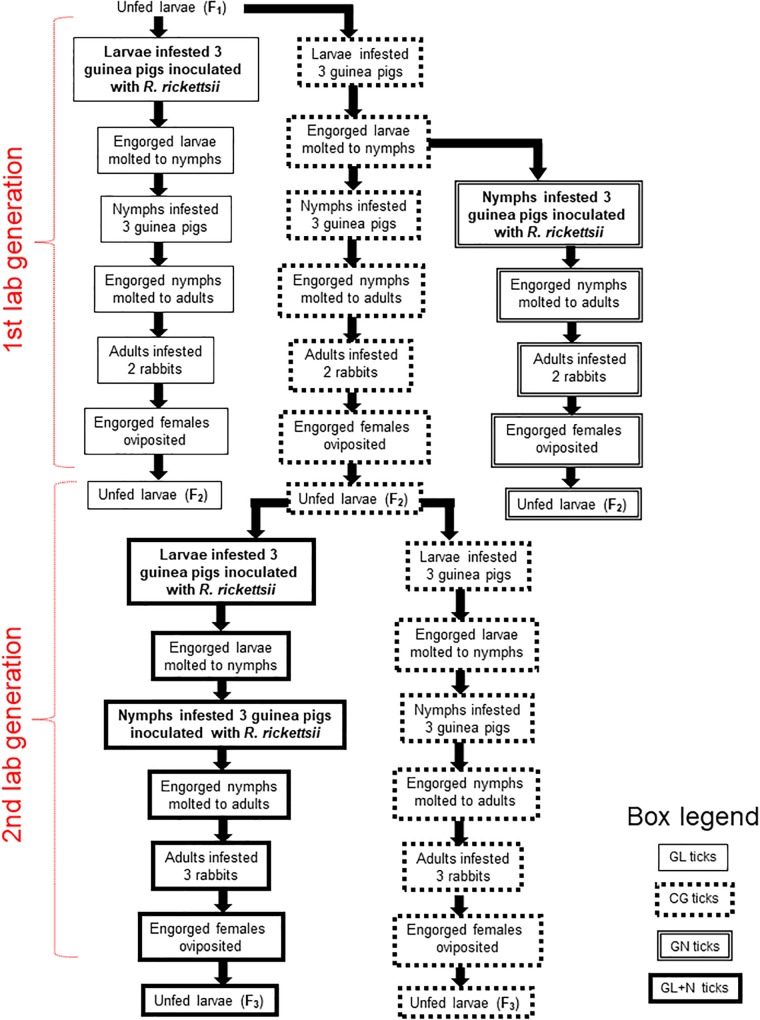
Diagram illustrating experimental procedures with each of the six colonies of *Amblyomma sculptum* ticks from F_1_ larvae to F_3_ larvae. GL and GN ticks were exposed to *Rickettsia rickettsii-*inoculated guinea pigs during the F_1_ larval and nymphal stages, respectively, and thereafter, they were reared through the F_2_ larval stage on susceptible animals (guinea pigs for larvae and nymphs; rabbits for adult ticks). GL + N ticks were dually exposed to *R. rickettsii-*inoculated guinea pigs, firstly during the F_2_ larval stage, secondly during the F_2_ nymphal stage, and then reared through the F_3_ larval stage on susceptible rabbits. CG ticks were the uninfected control group, always exposed to susceptible guinea pigs or rabbits in parallel to the infected groups.

### Experimental Procedures

Most of the experimental procedures used in this study were exactly the same that were used in a previous study at our laboratory, in which an *A. sculptum* population from Pedreira, state of São Paulo, was experimentally infected with *R. rickettsii* strain Taiaçu, previously isolated from the tick *A. aureolatum* (Soares et al., 2012). These procedures were adopted, so most of our results can be compared to the results reported by Soares et al. (2012), as presented below in the Discussion section.

In the present study, the strain Itu of *R. rickettsii* was used for experimental infection of five tick colonies: E-ITU, E-PIC, NE-PIS, NE-POC, and NE-GSV. The strain Pampulha of *R. rickettsii* was used for experimental infection of only the E-PAM tick colony. With these procedures, we had two tick colonies exposed to autochthone strains of *R. rickettsii* (E-ITU to strain Itu; E-PAM to strain Pampulha), and four tick colonies exposed to a non-autochthone strain of *R. rickettsii* (E-PIC, NE-PIS, NE-POC, and NE-GSV to strain Itu); in the latter case, one tick colony (E-PIC) was from a BSF-endemic area, while the remaining three tick colonies were from BSF non-endemic areas. In addition, NE-POC and NE-GSV were from highly preserved areas with rich biodiversity, whereas NE-PIS was from a highly anthropic area (Table 1).

Procedures of experimental infection of ticks with *R. rickettsii* and the follow up infestations, from F_1_ larvae to F_3_ larvae (or F_3_ nymphs for a single tick colony) were the same for each tick colony, and are summarized in Figure 1. In all cases, the initial exposition of ticks to *R. rickettsii* infection consisted of ticks (larvae or nymphs) feeding on *R. rickettsii-*infected guinea pigs. For this purpose, samples from our stock of cryopreserved guinea pig-infected organs were thawed at room temperature, crushed in a mortar with brain–heart infusion (BHI) and the resultant homogenate was used to inoculate guinea pigs intraperitoneally, as previously described (Soares et al., 2012). At the same day of guinea pig inoculation, these animals were infested with ticks. During the F_1_ generation, three experimental groups of ticks (GL, GN, and CG) were formed in each colony. GL and GN consisted of ticks that were exposed to *R. rickettsii-*inoculated guinea pigs during the F_1_ larval or nymphal stages, respectively, and thereafter they were reared through the F_2_ larval stage on susceptible hosts (guinea pigs or rabbits). CG was the uninfected control group, always exposed to susceptible, uninfected hosts (Figure 1).

In the second lab generation of the study, F_2_ larvae of the uninfected control group (CG) of each tick colony were exposed to *R. rickettsii-*inoculated guinea pigs, and the resultant nymphs were also exposed to *R. rickettsii-*inoculated guinea pigs, forming the GL + N group (ticks with dual exposure to *R. rickettsii-*inoculated guinea pigs). These ticks were reared through the F_3_ larval or nymphal stage, with ticks feeding on susceptible, uninfected hosts. In parallel, CG ticks were reared through the F_3_ larval stage, always exposed to susceptible, uninfected hosts (Figure 1).

### Guinea Pig and Rabbit Evaluation

All infested animals had their rectal temperature measured from 0 to 21 days post-inoculation or infestation (dpi). Guinea pigs were considered febrile when rectal temperature was >39.5°C (Monteiro, 1931); rabbits were considered febrile when rectal temperature was >40.0°C (Monteiro, 1933). The occurrence of scrotal reactions (edema, congestion, necrosis), typical of *R. rickettsii* acute infection in guinea pigs and rabbits (Monteiro, 1933), were annotated. In all infestations, approximately 0.5–1.0 mL of blood was obtained from each guinea pig or rabbit at days 0 and 21 dpi. Guinea pig blood samples were collected intra-cardiacally under anesthesia (20 mg/Kg xylazine + 20 mg/kg cetamin + 1 mg/kg acepromazin), while rabbit samples were collected through the central ear vein. Samples were centrifuged to obtain sera, which were tested for seroconversion to *R. rickettsii*, as described below. If any guinea pig or rabbit died before 21 dpi, it was subjected to necropsy, when we tried to obtain a blood sample through cardiac puncture. In addition, a lung sample was collected and evaluated by PCR for rickettsial infection, using the protocol described below.

### Molecular Analysis

Throughout the study, samples of unfed larvae, nymphs and adults, eggs, and engorged females at the end of oviposition, were tested by PCR for detection of rickettsial DNA. For this purpose, DNA was extracted from eggs (in pools of ≈50 eggs or individually), unfed larvae (in pools of ≈50 larvae or individually), and unfed nymphs by the boiling method, as previously described (Horta et al., 2005), and from unfed adult ticks or engorged females at the end of oviposition by the guanidine isothiocyanate-phenol technique (Sangioni et al., 2005). Guinea pig or rabbit lung fragments were submitted to DNA extraction using the DNeasy tissue Kit (Qiagen, Chatsworth, CA, United States). Blank tubes were always included in the DNA extraction procedures, as negative controls of this step.

Extracted DNA samples were tested by a Taqman real-time PCR assay targeting the rickettsial *gltA* gene, as described (Soares et al., 2012). The sensitivity of this PCR assay was determined to be one DNA copy of *R. rickettsii* (Labruna et al., 2004). Tick DNA samples that were shown to contain no rickettsial DNA by the real-time PCR described above were tested by a conventional PCR protocol targeting the tick mitochondrial 16S rRNA gene, as previously described (Mangold et al., 1998). If the tick sample yielded no product by this PCR, it was considered that DNA extraction was not successful, and the sample was discarded from the study. Random samples of F_1_ adult ticks, positive by the real-time PCR assay, were further tested using a conventional PCR assay targeting a 532-bp fragment of the rickettsial *ompA* gene, as described (Regnery et al., 1991); PCR products were DNA-sequenced and the resultant sequences were submitted to BLAST analysis^1^ in order to confirm the identity of the *Rickettsia* species.

### Serological Analysis

Guinea pig and rabbit blood serum samples were individually tested by the indirect immunofluorescence assay (IFA) using crude antigens derived from *R. rickettsii* strain Taiaçu, as previously described (Labruna et al., 2007). Each serum was diluted in two-fold increments with PBS from 1:64 to the endpoint titer (Labruna et al., 2007). A commercial fluorescein isothiocyanate-labeled goat anti-rabbit IgG (Sigma Diagnostics, St. Louis, MO, United States) or rabbit anti-guinea pig IgG (Sigma Diagnostics) was used. In each slide, a serum previously shown to be non-reactive (negative control) and a known reactive serum (positive control) were tested at the 1:64 dilution. These serum samples were from the study of Soares et al. (2012).

### Statistical Analyses

The proportions of ticks that successfully molted or oviposited fertile eggs from different groups, the proportion of *R. rickettsii-*infected ticks from different colonies, and fatality rates of inoculated guinea pigs were compared by using the chi-square test. Reproductive parameters of engorged females were compared between colonies/groups by using the *t*-test, after the Kolmogorov–Smirnov normality test. Analyses were performed using the program Epi Info^TM^ version 7 (chi-square) and Minitab^®^ 18.1 (*t*-Student). Significant differences were considered for *P* < 0.05.

### Phylogenetic Analyses of Ticks

In order to perform a comparative genetic analysis of *R. rickettsii-*infected and uninfected ticks of each colony, we generated partial sequences of the tick mitochondrial 16S rRNA gene and the tick second internal transcribed spacer (ITS2). Noteworthy, the uninfected tick samples were composed by adult ticks that were exposed to *R. rickettsii* infection (through feeding as larvae and/or nymphs on rickettsemic guinea pigs), but were shown to contain no rickettsial DNA when tested as unfed adults by the real-time PCR protocol described above.

From each tick colony, DNA of *R. rickettsii-*infected and uninfected ticks were submitted to two PCR protocols, one targeting a ≈460-bp fragment of the tick mitochondrial 16S rRNA gene (Mangold et al., 1998) and one targeting a ≈1,100-bp of the tick nuclear ribosomal region, which includes the entire ITS2, as previously described (Marrelli et al., 2007). All PCR products were treated with ExoSap (USB, Cleveland, OH, United States) and sequenced in an ABI automated sequencer (Applied Biosystems/Thermo Fisher Scientific, model ABI 3500 Genetic Analyzer, Foster City, CA, United States) with the same primers used for PCR. The consensus 16S rRNA sequences were aligned by using Clustal/W v.1.8.1 (Thompson et al., 1994), including the 16S rRNA sequence of *Amblyomma tonelliae* from Paraguay retrieved from GenBank (KF179349), which was used as the outgroup. A nucleotide identity matrix was calculated by using the Bioedit software v.7.0.5.3 (Hall, 1999). A phylogenetic tree was generated with the Maximum Likelihood criteria (Tamura 3-parameter model) for the 16S rRNA mitochondrial gene partial nucleotide sequences (410 sites on the dataset) and 1,000 bootstrap replicates using Mega X software (Kumar et al., 2018).

## Results

### Inoculated Guinea Pigs

In order to form the *R. rickettsii*-infected tick cohorts, larvae and nymphs of the six colonies were exposed to *R. rickettsii* by feeding on guinea pigs that had been inoculated with homogenates of *R. rickettsii-*infected guinea pig organs. A total of 72 guinea pigs were inoculated, which all but one developed fever that started 3–8 dpi (median: 4; mean: 3.9 ± 0.9), and lasted for 3–11 days (median: 5; mean: 5.4 ± 2.0) (Supplementary Tables S1–S12). Ticks that had fed on the only non-febrile-inoculated guinea pig (guinea pig G.P.78) were discarded from the study (Supplementary Table S6). Rickettsial infection was confirmed in all febrile guinea pigs by seroconversion to *R. rickettsii* at 21 dpi (endpoint titers ranging from 16,384 to 65,536; median: 32,768) or by detection of rickettsial DNA in a lung sample collected from those guinea pigs that died during the febrile period. For instance, 53 out of 71 guinea pigs died during the febrile period (Supplementary Tables S1–S12), resulting in an overall fatality rate of 74%. Considering the two *R. rickettsii* strains separately, fatality rates were 75% (45/60) for strain Itu, and 72% (8/11) for strain Pampulha (*P* > 0.05). Larval or nymphal feeding periods lasted from 3 to 10 days, with most of the engorged ticks detaching between 4 and 7 dpi. Since larval and nymphal infestations were performed at the same day of guinea pig inoculation, tick feeding period overlapped with the febrile period, a condition that certifies that these ticks fed during the rickettsemic period; hence, they were exposed to *R. rickettsii*.

### Rickettsial Infection in GL Ticks

The GL ticks consisted of F_1_ larvae initially exposed to *R. rickettsii* by feeding on rickettsemic guinea pigs, and thereafter, reared until F_2_ unfed larvae by feeding on susceptible hosts. After F_1_ acquisition larval feeding, rickettsial infection rates of F_1_ nymphs (transstadial perpetuation of *R. rickettsii* from larvae to nymphs) varied from 0 to 16% among the six tick colonies, with higher rates for the two tick colonies that were exposed to autochthone *R. rickettsii* strains, namely E-ITU and E-PAM (Table 2). Infestation of these F_1_ nymphs on susceptible guinea pigs resulted in successful rickettsial transmission (fever followed by death or seroconversion with high endpoint titers) in all three guinea pigs exposed to E-ITU and E-PAM nymphs, only one guinea pig exposed to NE-PIS or NE-GSV, and none of the guinea pigs exposed to E-PIC and NE-POC (Table 3). Transstadial perpetuation of *R. rickettsii* from F_1_ nymphs to adults were observed by DNA detection only in E-ITU and E-PAM ticks (33 and 47%, respectively), which were infected with autochthone *R. rickettsii* strains (Table 2). Infestation of F_1_ adults on susceptible rabbits resulted in successful rickettsial transmission (fever followed by death or seroconversion with high endpoint titers) in all two rabbits exposed to E-ITU ticks, and one out of two rabbits exposed to E-PAM, NE-PIS, and NE-GSV adults, and none of the two rabbits exposed to either E-PIC or NE-POC (Table 3). Transovarial transmission of *R. rickettsii* was observed by DNA detection in engorged females of only the E-ITU, E-PAM, and NE-PIS tick colonies. In these cases, the transovarial transmission rate (no. females with PCR-positive eggs or larvae/no. PCR-positive females at the end of oviposition × 100) varied from 12 to 18% (Table 2). For these infected females with rickettsial transovarial transmission, we calculated the filial infection rate (No. infected eggs or larvae/No. tested eggs or larvae × 100), which was 100% for either E-ITU or E-PAM ticks (infected with autochthone *R. rickettsii* strains), and 0–10% for NE-PIS ticks (infected with non-autochthone *R. rickettsii* strain) (Supplementary Table S13). The transovarial acquisition infection of E-PAM F_2_-infected larvae was confirmed by allowing part of these larvae to feed on a guinea pig, which became ill and seroconverted to *R. rickettsii* (guinea pig G.P. 73 in Supplementary Table S5).

**Table 2:** Tick-*Rickettsia rickettsii* acquisition (feeding on rickettsemic guinea pig) and maintenance (transstadial perpetuation and transovarial transmission).

Tick colony	Transstadial perpetuation: larvae to nymphs^*a*^			Transstadial perpetuation: nymphs to adults^*b*^			Transovarial transmission rate^*c*^		
	GL^*d*^	GN	GL + N^*d*^	GL	GN	GL + N	GL	GN	GL + N
E-ITU	4/25 (16)^a^	–	3/36 (8)^a^	5/15 (33)	0/15 (0)	9/15 (60)	2/16 (12)	3/11 (27)	4/20 (20)
E-PIC	1/40 (3)^a,b^	–	3/30 (10)^a^	0/10 (0)	0/30 (0)	0/30 (0)	0 (0/0)	0 (0/0)	0/2 (0)
E-PAM	3/36 (8)^a,b^	–	9/22 (41)^b,c^	7/15 (47)	10/15 (67)	5/15 (33)	1/7 (14)	1/6 (17)	0/4 (0)
NE-PIS	1/55 (2)^b^	–	0/10 (0)^a^	0/25 (0)	3/28 (11)	20/30 (67)	2/11 (18)	1/15 (7)	3/19 (16)
NE-POC	1/80 (1)^b^	–	0/15 (0)^a^	0/20 (0)	0/15 (0)	3/15 (20)	0 (0/0)	0 (0/0)	1/8 (13)
NE-GSV	0/48 (0)^b^	–	5/31 (16)^a,c^	0/16 (0)	4/22 (18)	7/30 (23)	0/5 (0)	1/6 (17)	3/11 (27)

*GL, R. rickettsii-acquisition feeding as larvae; GN, R. rickettsii-acquisition feeding as nymphs; and GL + N, dual R. rickettsii acquisition-feeding, as larvae and as nymphs. ^a^No. PCR-positive unfed nymphs/no. unfed nymphs tested (% infection). ^b^No. PCR-positive unfed adults/no. unfed adults tested (% infection). ^c^No. females with PCR-positive eggs or larvae/no. PCR-positive females at the end of oviposition (% transovarial transmission). ^d^Different letters in the same column mean significantly different infection rates (P < 0.05).*

**Table 3:** Results of rickettsial transmission by ticks (vector competence) of six colonies of *Amblyomma sculptum* that were allowed to feed on susceptible hosts (guinea pigs for larvae and nymphs; rabbits for adults).

Tick colony	No. hosts that became infected by *R. rickettsii*/no. infested hosts^*a*^									
	GL ticks			GN ticks			GL + N ticks			Total (%)
	F_1_ nymphs	F_1_ adults	F_2_ larvae	F_1_ adults	F_2_ larvae	F_2_ nymphs	F_2_ adults	F_3_ larvae	F_3_ nymphs	
E-ITU	3/3	2/2	–	2/2	–	–	3/3	2/2	1/1	13/13 (100)
E-PIC	0/3	0/2	–	0/2	–	–	1/3	–	–	1/10 (10)
E-PAM	3/3	1/2	1/1	1/2	1/1	–	0/1	–	–	7/10 (70)
NE-PIS	1/3	1/2	–	2/2	1/1	1/1	2/3	–	–	8/12 (67)
NE-POC	0/3	0/2	–	0/2	–	–	3/3	–	–	3/10 (30)
NE-GSV	1/3	1/2	–	1/2	–	–	2/3	2/3	–	7/13 (54)

*Previous to these infestations, Rickettsia rickettsii-acquisition feedings were performed on rickettsemic guinea pigs that were infested with F_1_ larvae (GL ticks), F_1_ nymphs (GN ticks) and F_2_ larvae and nymphs (GL + N ticks). GL, R. rickettsii-acquisition feeding as larvae; GN, R. rickettsii-acquisition feeding as nymphs; and GL + N, dual R. rickettsii acquisition-feeding, as larvae and as nymphs. ^a^Hosts were considered infected by R. rickettsii when they became febrile after tick infestation, and the infection was confirmed by seroconversion with high endpoint titers to R. rickettsii or by detection of rickettsial DNA in their lungs if they died during the febrile period. −, not done.*

### Rickettsial Infection in GN Ticks

The GN ticks consisted of F_1_ nymphs initially exposed to *R. rickettsii* by feeding on rickettsemic guinea pigs, and thereafter, reared until F_2_ unfed larvae or nymphs by feeding on susceptible hosts. After F_1_ acquisition nymphal feeding, rickettsial infection rates of F_1_ adults (transstadial perpetuation of *R. rickettsii* from nymphs to adults) were observed by DNA detection in ticks of only three tick colonies, 67% for E-PAM, 11% for NE-PIS, and 18% for NE-GSV (Table 2). Infestation of these F_1_ adults on susceptible rabbits resulted in successful rickettsial transmission (fever followed by death or seroconversion with high endpoint titers) in all two rabbits exposed to either E-ITU, E-PAM or NE-PIS ticks, and one out of two rabbits exposed to NE-GSV adults, and none of the two rabbits exposed to either E-PIC or NE-POC (Table 3). Transovarial transmission of *R. rickettsii* was observed by DNA detection in engorged females of the E-ITU, E-PAM, NE-PIS, and NE-GSV tick colonies. In these cases, the transovarial transmission rate varied from 7 to 27% (Table 2). For these infected females with rickettsial transovarial transmission, the filial infection rates were 90–100% for E-ITU, 60% for E-PAM, 10–50% for NE-PIS, and 70% for NE-GSV (Supplementary Table S13). The transovarial acquisition infection of either E-PAM or NE-PIS F_2_-infected larvae was confirmed by allowing part of these larvae to feed on guinea pigs, which became ill and seroconverted to *R. rickettsii* (guinea pigs G.P.74 in Supplementary Table S5, and G.P.102 in Supplementary Table S7). Moreover, NE-PIS F_2_ nymphs (molted from the engorged larvae that had fed on G.P.102) were also able to transmit *R. rickettsii* to guinea pig G.P.103 (Tables 3 and Supplementary Table S7).

### Rickettsial Infection in GL + N Ticks

The GL + N ticks consisted of F_2_ uninfected ticks that were dually exposed to *R. rickettsii* by feeding on rickettsemic guinea pigs during both larval and nymphal feeding, and thereafter, reared until F_3_ larvae or nymphs by feeding on susceptible hosts. After F_2_ acquisition nymphal feeding, rickettsial infection rates of F_2_ nymphs (transstadial perpetuation of *R. rickettsii* from larvae to nymphs) were observed by DNA detection in ticks of four tick colonies, 41% for E-PAM, 16% for NE-GSV, 10% for E-PIC, and 8% for E-ITU ticks (Table 2). After the second acquisition feeding (F_2_ nymphs on rickettsemic guinea pigs), rickettsial infection rates of F_2_ adults (transstadial perpetuation of *R. rickettsii* from nymphs to adults) were observed in ticks of five out of the six tick colonies, 67% for NE-PIS, 60% for E-ITU, 33% for E-PAM, 23% for NE-GSV, and 20% for NE-POC ticks; rickettsial infection was not detected in any of the E-PIC adult ticks (Table 2). Infestation of these F_2_ adults on susceptible rabbits resulted in successful rickettsial transmission (fever followed by death or seroconversion with high endpoint titers) in all three rabbits exposed to either E-ITU or NE-POC ticks, two out of three rabbits exposed to either NE-PIS or NE-GSV ticks, and one out of three rabbits exposed to E-PIC adults. Rickettsial transmission was not observed in any of the two rabbits exposed to either E-PIC or NE-POC, and in the one exposed to E-PAM ticks (Table 3). Transovarial transmission of *R. rickettsii* was observed in engorged females of four colonies, with transovarial transmission rates of 27% for NE-GSV, 20% for E-ITU, 16% for NE-PIS, and 13% for NE-POC. There was no transovarial transmission in E-PIC and E-PAM ticks (Table 2). For the infected females with rickettsial transovarial transmission, the filial infection rates were 30–100% for NE-GSV, 0–100% for E-ITU, 50–100% for NE-PIS, and 100% for NE-POC (Supplementary Table S13). The transovarial acquisition infection of either E-ITU or NE-GSV F_2_-infected larvae was confirmed by allowing part of these larvae to feed on guinea pigs, which became ill and seroconverted to *R. rickettsii* (guinea pigs G.P.28 and G.P.29 in Table S2, and G.P.171 and G.P.172 in Table S12). Moreover, E-ITU F_2_ nymphs (molted from the engorged larvae that had fed on G.P.29) were also able to transmit *R. rickettsii* to guinea pig G.P.30 (Tables 3 and Supplementary Table S2).

### CG Ticks and Additional Molecular Tests

Each of the six tick colonies had its own CG group, which consisted of uninfected ticks that were reared in parallel to each of the infected groups, always exposed to susceptible guinea pigs or rabbits. During all infestations from F_1_ larvae to F_2_ adults, all animals remained afebrile and did not seroconvert to *R. rickettsii*. Moreover, larvae, nymphs and adult ticks were always negative by PCR, indicating absence of rickettsiae in this tick control group (Supplementary Tables S1–S12).

All *Rickettsia-*negative samples from the six experimental groups yielded amplicons by the PCR assay targeting the tick 16S rRNA gene, indicating that DNA extraction was successful. The only few exceptions, five tick samples that failed to amplify the tick 16S rRNA gene, were discarded from the study. Random samples of two *R. rickettsii-*infected F_1_ adult ticks per colony yielded *ompA* amplicons that generated DNA sequences 100% identical to the sequence of *R. rickettsii* strain Taiaçu from GenBank (KU321853).

### Tick Molting Success and Reproductive Performance

Molting success rates of engorged larvae to nymphs were higher for CG (uninfected ticks) than for GL or GL + N (exposed to *R. rickettsii*) ticks in all six tick colonies, with statistically significant differences (*P* < 0.05) in most of the cases (Table 4). A similar trend was observed for engorged nymphs, which displayed significantly higher (*P* < 0.05) molting success for CG ticks in most of the times when compared to GL, GN or GL + N ticks (Table 4). Hence, exposure to *R. rickettsii* infection caused higher mortality rates of engorged larvae and nymphs of all six tick colonies.

**Table 4:** Molting success according to *Amblyomma sculptum* tick generations (F_1_ or F_2_), tick stages (larvae or nymph) and experimental groups (CG, GL, GN, and GL + N).

Tick colonies	No. ticks that molted/no. engorged ticks (% molting success)^*a*^								
	F_1_ larvae^*b*^		F_1_ nymphs			F_2_ larvae^*b*^		F_2_ nymphs	
	CG	GL	CG	GL	GN	CG	GL + N	CG	GL + N
E-ITU	273/300 (91)^a^	126/300 (42)^b^	242/242 (100)^a^	190/276 (69)^b^	175/234 (75)^b^	243/300 (81)^a^	166/300 (55)^b^	246/257 (96)^a^	254/289 (88)^b^
E-PIC	267/300 (89)^a^	264/300 (88)^a^	255/256 (99)^a^	258/270 (96)^b^	272/272 (100)^a^	274/300 (91)^a^	159/300 (53)^b^	278/280 (99)^a^	253/269 (94)^b^
E-PAM	208/300 (69)^a^	199/300 (66)^a^	237/242 (98)^a^	240/283 (86)^b^	271/280 (97)^a^	283/300 (94)^a^	171/300 (57)^b^	129/145 (89)^a^	106/159 (67)^b^
NE-PIS	260/300 (87)^a^	247/300 (82)^a^	204/204 (100)^a^	232/248 (94)^b^	209/210 (99)^a^	283/300 (94)^a^	176/300 (59)^b^	192/192 (100)^a^	212/212 (100)^a^
NE-POC	217/300 (72)^a^	191/300 (64)^b^	202/207 (98)^a^	186/204 (91)^b^	160/171 (94)^a,b^	263/300 (88)^a^	186/300 (62)^b^	247/260 (95)^a^	236/245 (96)^a^
NE-GSV	294/300 (98)^a^	272/300 (91)^b^	173/183 (95)^a^	272/278 (98)^a^	173/222 (78)^b^	285/300 (95)^a^	193/300 (64)^b^	214/216 (99)^a^	180/225 (80)^b^

*CG, uninfected control group, never exposed to R. rickettsii infection; GL, R. rickettsii-acquisition feeding as larvae; GN, R. rickettsii-acquisition feeding as nymphs; and GL + N, dual R. rickettsii acquisition-feeding, as larvae and as nymphs. ^a^Different letters in the same line for the same tick generation and stage mean significantly different molting success values (P < 0.05). ^b^Due to the high number of engorged larvae recovered from guinea pigs, molting success was observed in a sample of 300 viable engorged larvae from each generation of each tick colony.*

Overall, the reproductive performance of engorged females of the uninfected ticks tended to be higher than those of the *R. rickettsii-*infected ticks, as demonstrated by higher engorged female weight, egg mass weight, CEI, and % egg hatching values of the uninfected females (Supplementary Tables S14–S19). However, only in a few cases these values were significantly different (*P* < 0.05) between uninfected and infected females of the same tick group, as for example, the engorged female weight and % egg hatching values in the GL group of E-ITU ticks (Supplementary Table S14), engorged female weight and CEI values in the GL + N group of E-PIC ticks (Supplementary Table S15), the CEI values in the GL group of E-PAM ticks (Supplementary Table S16), and the % egg hatching values in the GL group of NE-PIS ticks (Supplementary Table S17) and the GL + N group of NE-POC ticks (Supplementary Table S18).

### Tick Molecular and Phylogenetic Analyses

**FIGURE 2:**
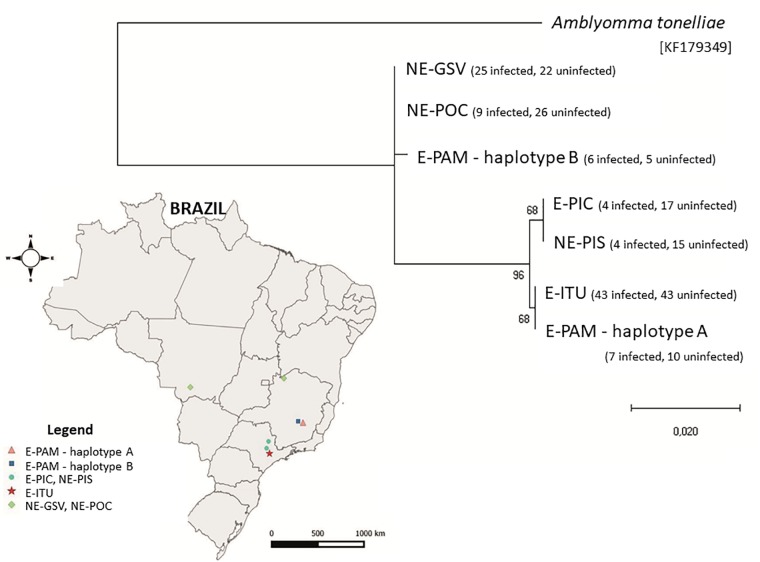
Maximum likelihood tree based on the Tamura 3-parameter model for the 16S rRNA mitochondrial gene partial nucleotide sequences of six tick colonies (E-ITU, E-PIC, E-PAM, NE-PIS, NE-POC, and NE-GSV) of *Amblyomma sculptum* (410 sites on the dataset). The sequence of *Amblyomma tonelliae* from Paraguay (KF179349) was used as outgroup. The numbers at each node are bootstrap values greater than 60% from 1,000 replicates. The tree is drawn to scale, with branch lengths measured in the number of substitutions per site. The map of Brazil indicates the geographical location of each of the seven *A. sculptum* haplotypes from the tree. The numbers of sequences obtained for infected and uninfected ticks for each haplotype are shown within parentheses.

Regarding the mitochondrial 16S rRNA gene, we generated partial sequences from a total of 98 *R. rickettsii-*infected ticks (4–43 sequences from each of the six colonies) and from 138 uninfected ticks (15–43 ticks from each of the six colonies) (Supplementary Table S20). Ticks of each colony yielded a single haplotype, except for the E-PAM ticks, which yielded two distinct haplotypes (A and B). E-PIC and NE-PIS ticks shared the same haplotype, as also did NE-GSV and NE-POC ticks, and also E-ITU and part of the E-PAM ticks; the other part of E-PAM yielded a unique haplotype (Supplementary Table S21). Despite these inter-colony differences, *R. rickettsii-*infected and uninfected ticks of the same colony shared a single haplotype. Even among E-PAM ticks (the only colony that generated more than one haplotype), each of the two haplotypes were shared by both infected and uninfected ticks. Differences among the seven haplotypes of *A. sculptum* ranged from 0% (between the above identical haplotypes) to 3.3% [between E-PAM (haplotype B) and either NE-PIS or E-PIC]. E-PAM haplotypes A and B differed by 3% (Supplementary Table S21). The phylogenetic tree containing these seven 16S rRNA haplotypes shows two major clusters, one containing E-ITU, E-PAM (haplotype A), E-PIC and NE-PIS ticks, which segregated under high bootstrap support (96%) from the other clade that contains NE-GSV, NE-POC, and E-PAM (haplotype B) (Figure 2).

Regarding the nuclear ITS2 nuclear gene, we generated partial sequences of 557-bp from a total of 77 *R. rickettsii-*infected ticks (3–40 sequences from each of the six colonies) and from 112 uninfected ticks (8–40 ticks from each of the six colonies). All these sequences were identical to each other, and when submitted to BLAST analysis, they were 100% identical to several *A. sculptum* sequences from Brazil (KU169885-KU169888).

## Discussion

Exposure of ticks of six geographically different populations of *A. sculptum* to *R. rickettsii* acquisition feeding and transmission revealed different susceptibilities among the six tick populations. After larval acquisition feeding (GL ticks), these differences were clearly in favor of higher infection rates for the two tick colonies that were exposed to their autochthone *R. rickettsii* strain, namely strain Itu for E-ITU ticks, and strain Pampulha for E-PAM ticks. Rickettsial infection rates in F_1_ unfed nymphs of these two colonies were significantly higher than those observed in the other four tick colonies, which were exposed to *R. rickettsii* strain Itu. These differences among GL ticks were even more remarkable for the F_1_ adult stage, as shown by 33 and 47% rickettsial detection in unfed adults of E-ITU and E-PAM ticks, in contrast to 0% for the remaining tick colonies (Table 2). The same trend was somewhat observed when *R. rickettsii* acquisition feeding occurred during the nymphal stage (GN ticks) or during both larval and nymphal feeding (GL + GN ticks), as shown by generally higher rickettsial infection rates among E-PAM and E-ITU ticks. While comparable infection rates were sometimes observed in ticks of the other colonies, it is noteworthy that 0% infection rates were observed several times for E-PIC, NE-PIS, NE-POC and NE-GSV ticks. In addition, highest rates of transovarial transmission of *R. rickettsii* also included E-PAM and E-ITU ticks, while 0% rates were more frequent on the other tick colonies (Table 2). These results were corroborated by the vector competence infestations, which resulted in more events of successful transmission of *R. rickettsii* when susceptible hosts were infested by E-PAM and E-ITU ticks; i.e., 70–100% successful transmission versus 10–67% (Table 3). All together, these results indicate that the susceptibility of *A. sculptum* to *R. rickettsii* varies among different tick populations, with a clear bias for higher susceptibility to an autochthone *R. rickettsii* strain that has already coevolved with a tick population for some time.

Soares et al. (2012) evaluated the susceptibility of an *A. sculptum* population by an *A. aureolatum*-derived strain of *R. rickettsii*, using the same type of protocols of the present study. Their results for the GL group (acquisition feeding during the larva stage) were comparable with our results for the tick colonies that were exposed to a non-autochthone *R. rickettsii* strain, although their GN group (acquisition feeding during the nymphal stage) displayed infection rates similar or superior to all GN groups of the present study. On the other hand, most of the transovarial acquisition infection-larvae of the study of Soares et al. (2012) failed to transmit *R. rickettsii* to guinea pigs, contrasting to the high vector competence of the transovarial acquisition infection-larvae of the present study. These results reinforce our above statement that suggests the existence of some adaptive interaction of *R. rickettsii* strains with its natural tick populations. Interestingly, our molecular analyses of two tick genes (16S rRNA and ITS2) showed that ticks of the two most *R. rickettsii-*susceptible populations of the present study (E-PAM and E-ITU) shared the same 16S rRNA haplotype (Figure 2), suggesting a genetic role in the tick-*R. rickettsii* interaction. However, further studies are needed to investigate this interaction more deeply, which could also be affected by tick endosymbionts, as for example, bacteria of the genera *Coxiella*, which have been reported to infect some *A. sculptum* (reported as *A. cajennense*) populations (Machado-Ferreira et al., 2011; Duron et al., 2015).

Regardless of the different susceptibilities of *A. sculptum* populations to *R. rickettsii*, Soares et al. (2012) and the present study clearly demonstrated that this tick species is partially refractory to *R. rickettsii* infection, in conjunction with relatively low transovarial transmission rates, always <50%. In addition, our results demonstrated that the *R. rickettsii* infection induces higher mortality of engorged larvae and nymphs, and tend to reduce the reproductive fitness of engorged females; this later impairment was also demonstrated by Soares et al. (2012). While these findings have been used to explain the low *R. rickettsii-*infection rates of *A. sculptum* under natural conditions, usually <1% (Krawczak et al., 2014; Labruna et al., 2017), they indicate that an *A. sculptum* population should not be able to sustain a *R. rickettsii* infection for successive tick generations without the creation of new cohorts of infected ticks via horizontal transmission on vertebrate rickettsemic hosts (amplifying hosts). In this case, capybaras have been incriminated as the main amplifying host of *R. rickettsii* for *A. sculptum* ticks in most of the BSF-endemic areas of Brazil (Souza et al., 2009; Polo et al., 2017). Finally, despite the ubiquitous distribution of *A. sculptum* in southeastern and central-western Brazil (Martins et al., 2016), most of the populations of this tick species are devoid of *R. rickettsii* infection (Sangioni et al., 2005; Pacheco et al., 2007, 2009). This scenario might be related to two major factors: (i) insufficient numbers of susceptible amplifying hosts; and (ii) lower susceptibilities of many tick populations. While the first factor has been demonstrated by mathematical models (Polo et al., 2017), the second is highlighted by the results observed in the present study.

## Data Availability

The raw data supporting the conclusions of this manuscript will be made available by the authors, without undue reservation, to any qualified researcher.

## Ethics Statement

This study has been approved by the Institutional Animal Care and Use Committee (IACUC) of the Faculty of Veterinary Medicine of the University of São Paulo (protocols 3104/2013 and 5948070314). Field capture of ticks was authorized by the Brazilian Ministry of the Environment (permit SISBIO Nos. 43259-1 and 11459-1).

## Author Contributions

MG and ML designed the experiments. MG, AR-H, LB, and FK performed the experiments. MG, FG, and ML analyzed the data and wrote the manuscript. All authors read and approved the final manuscript.

## Conflict of Interest Statement

The authors declare that the research was conducted in the absence of any commercial or financial relationships that could be construed as a potential conflict of interest.

## Footnotes

1 www.ncbi.nlm.nih.gov/blast
