# Supplementary material for: Comparative Susceptibility of Different Populations of Amblyomma sculptum to Rickettsia rickettsii
Source: Front Physiol. 2019 May 28;10:653. doi: 10.3389/fphys.2019.00653 (PMC6546895; doi:10.3389/fphys.2019.00653)
Supplement: Supplementary file 1 [file Data_Sheet_1.docx]

**Table S1.** Results of infestations with the first generation (F_1_) of an *Amblyomma sculptum* tick colony from Itu Municipality (E-ITU), which was divided into three groups: GL (exposed to *Rickettsia rickettsii-*inoculated guinea pigs during the larval stage), GN (exposed to *R. rickettsii-*inoculated guinea pigs during the nymphal stage), and GC (uninfected control group, never exposed to infected hosts).

| **Host code** | **Experimental condition**  **(tick group)** | **No. PCR-positive ticks / No. unfed ticks tested (%)** | **No. ticks that engorged** | **Fever period (dpi)** | **Scrotal reactions (dpi)** | **Death (dpi)** | **Serological titer to *R. rickettsii ^a^*** |
| --- | --- | --- | --- | --- | --- | --- | --- |
| G.P.1 | Infested with F_1_ larvae (GC) | 0/21 (0) *^b^* | 1,975 | no | no | no | <64 |
| G.P.2 |  |  | 102 | no | no | no | <64 |
| G.P.3 |  |  | 1,846 | no | no | no | <64 |
| G.P.4 | Inoculated with *R. rickettsii* and infested with F_1_ larvae (GL) | 0/21 (0) *^b^* | 808 | 4 - 6 | 5 | 7 *^d^* | <64 |
| G.P.5 |  |  | 940 | 3 - 10 | 5 | 13 *^d^* | <64 |
| G.P.6 |  |  | 1,160 | 3 - 7 | 4 | 9 *^d^* | <64 |
| G.P.7 | Infested with F_1_ nymphs (GC) | 0/10 (0) | 86 | no | no | no | <64 |
| G.P.8 |  |  | 86 | no | no | no | <64 |
| G.P.9 |  |  | 70 | no | no | no | <64 |
| G.P.10 | Infested with F_1_ nymphs (GL) | 4/25 (16) | 94 | 8 - 11 | no | 13 *^d^* | 128 |
| G.P.11 |  |  | 82 | 6 - 10 | no | 11 *^d^* | 128 |
| G.P.12 |  |  | 100 | 8 - 11 | no | 13 *^d^* | 256 |
| G.P.13 | Inoculated with *R. rickettsii* and infested with F_1_ nymphs (GN) | 0/10 (0) | 72 | 3 - 6 | 6 | 7 *^d^* | not done |
| G.P.14 |  |  | 75 | 5 - 7 | 7 | 9 *^d^* | not done |
| G.P.15 |  |  | 87 | 5 - 8 | no | 10 *^d^* | not done |
| R.1 | Infested with F_1_ adults (GC) | 0/10 (0) | 7 *^c^* | no | no | no | <64 |
| R.2 |  |  | 7 *^c^* | no | no | no | <64 |
| R.3 | Infested with F_1_ adults (GL) | 5/15 (33) | 11 *^c^* | 8 - 11 | 10 | 14 *^d^* | 1024 |
| R.4 |  |  | 11 *^c^* | 10 - 12 | no | no | 2048 |
| R.5 | Infested with F_1_ adults (GN) | 0/15 (0) | 8 *^c^* | 6 - 10 | 10 | 13 *^d^* | 1024 |
| R.6 |  |  | 13 *^c^* | 6 - 8 | 10 | 12 *^d^* | 128 |

G.P.: guinea pig; R.: rabbit; dpi: days post inoculation or infestation.

*^a^* If guinea pig died, its blood was collected at the death day in order to be tested by serology (indirect imunofluorescence assay with *R. rickettsii* antigens); if the guinea pig did not die, its blood was collected at 21 dpi.

*^b^* Refers to number of tick pools.

*^c^* Refers to the number of engorged females (it does not include male ticks that fed with females).

*^d^* The lung of this animal was collected at necropsy and was shown to contain rickettsial DNA by PCR.

**Table S2.** Results of infestations with the second (F_2_) and third (F_3_) generations of an *Amblyomma sculptum* tick colony from Itu Municipality (E-ITU), which was divided into two groups: GL+N (dually exposed to *Rickettsia rickettsii*-inoculated guinea pigs, firstly during the F_1_ larval stage, secondly during the F_1_ nymphal stage), and GC (uninfected control group, never exposed to infected hosts).

| **Host code** | **Experimental condition**  **(tick group)** | **No. PCR-positive ticks / No. unfed ticks tested (%)** | **No. ticks that engorged** | **Fever period (dpi)** | **Scrotal reactions (dpi)** | **Death (dpi)** | **Serological titer to *R. rickettsii ^a^*** |
| --- | --- | --- | --- | --- | --- | --- | --- |
| G.P.16 | Infested with F_2_ larvae (GC) | 0/14 (0) *^b^* | 632 | no | no | no | <64 |
| G.P.17 |  |  | 509 | no | no | no | <64 |
| G.P.18 |  |  | 1,402 | no | no | no | <64 |
| G.P.19 | Inoculated with *R. rickettsii* and infested with F_2_ larvae (GL+N) | 0/14 (0) *^b^* | 1,284 | 3 - 8 | 7 | 10 *^d^* | 256 |
| G.P.20 |  |  | 917 | 3 - 11 | 8 | no | 32,768 |
| G.P.21 |  |  | 771 | 4 - 7 | 6 | 9 *^d^* | 256 |
| G.P.22 | Infested with F_2_ nymphs (GC) | 0/15 (0) | 70 | no | no | no | <64 |
| G.P.23 |  |  | 92 | no | no | no | <64 |
| G.P.24 |  |  | 95 | no | no | no | <64 |
| G.P.25 | Inoculated with *R. rickettsii* and infested with F_2_ nymphs (GL+N) | 3/36 (8) | 99 | 8 - 11 | N.A. | 13 *^d^* | 128 |
| G.P.26 |  |  | 94 | 4 - 10 | 6 | no | 16,384 |
| G.P.27 |  |  | 96 | 4 - 6 | 6 | 9 *^d^* | 512 |
| R.7 | Infested with F_2_ adults (CG) | 0/10 (0) | 6 *^c^* | no | no | no | <64 |
| R.8 |  |  | 5 *^c^* | no | no | no | <64 |
| R.9 |  |  | 8 *^c^* | no | no | no | <64 |
| R.10 | Infested with F_2_ adults (GL+N) | 9/15 (60) | 9 *^c^* | 9 - 13 | 14 | no | 32,768 |
| R.11 |  |  | 4 *^c^* | 8 - 14 | 12 | no | 32,768 |
| R.12 |  |  | 9 *^c^* | 8 - 12 | 12 | no | 32,768 |
| G.P.28 | Infested with F_3_ larvae (GL+N) | 10/10 (100) | 16 | 6 - 11 | 8 | 13 | 4,096 |
| G.P.29 |  | 10/10 (100) | 99 | 7 - 12 | 8 | no | 2,048 |
| G.P.30 | Infested with F_3_ nymphs (GL+N) | 5/5 (100) | 22 | 7 - 12 | 11 | no | 4,096 |

G.P.: guinea pig; R.: rabbit; dpi: days post inoculation or infestation.

N.A. not applicable because the animal host was a female.

*^a^* If guinea pig died, its blood was collected at the death day in order to be tested by serology (indirect imunofluorescence assay with *R. rickettsii* antigens); if the guinea pig did not die, its blood was collected at 21 dpi.

*^b^* Refers to number of tick pools.

*^c^* Refers to the number of engorged females (it does not include male ticks that fed with females).

*^d^* The lung of this animal was collected at necropsy and was shown to contain rickettsial DNA by PCR.

**Table S3.** Results of infestations with the first generation (F_1_) of an *Amblyomma sculptum* tick colony from Piracicaba Municipality (E-PIC), which was divided into three groups: GL (exposed to *Rickettsia rickettsii-*inoculated guinea pigs during the larval stage), GN (exposed to *R. rickettsii-*inoculated guinea pigs during the nymphal stage), and GC (uninfected control group, never exposed to infected hosts).

| **Host code** | **Experimental condition**  **(tick group)** | **No. PCR-positive ticks / No. unfed ticks tested (%)** | **No. ticks that engorged** | **Fever period (dpi)** | **Scrotal reactions (dpi)** | **Death (dpi)** | **Serological titer to *R. rickettsii ^a^*** |
| --- | --- | --- | --- | --- | --- | --- | --- |
| G.P.31 | Infested with F_1_ larvae (GC) | 0/12 (0) *^b^* | 952 | no | no | no | <64 |
| G.P.32 |  |  | 1,156 | no | no | no | <64 |
| G.P.33 |  |  | 1,137 | no | no | no | <64 |
| G.P.34 | Inoculated with *R. rickettsii* and infested with F_1_ larvae (GL) | 0/12 (0) *^b^* | 970 | 4 - 6 | 5 | 8 *^d^* | not done |
| G.P.35 |  |  | 1,077 | 3 - 8 | 6 | no | 32,768 |
| G.P.36 |  |  | 888 | 5 - 8 | 7 | no | 16,384 |
| G.P.37 | Infested with F_1_ nymphs (GC) | 0/15 (0) | 89 | no | no | no | <64 |
| G.P.38 |  |  | 81 | no | no | no | <64 |
| G.P.39 |  |  | 86 | no | no | no | <64 |
| G.P.40 | Infested with F_1_ nymphs (GL) | 1/40 (3) | 83 | no | no | no | 64 |
| G.P.41 |  |  | 95 | no | no | no | <64 |
| G.P.42 |  |  | 92 | no | no | no | <64 |
| G.P.43 | Inoculated with *R. rickettsii* and infested with F_1_ nymphs (GN) | 0/15 (0) | 84 | 3 - 10 | no | no | 32,768 |
| G.P.44 |  |  | 93 | 4 - 11 | 7 | no | 16,384 |
| G.P.45 |  |  | 95 | 4 - 11 | 7 | 10 *^d^* | 16,384 |
| R.13 | Infested with F_1_ adults (GC) | 0/10 (0) | 11 *^c^* | no | no | no | <64 |
| R.14 |  |  | 13 *^c^* | no | no | no | <64 |
| R.15 | Infested with F_1_ adults (GL) | 0/10 (0) | 10 *^c^* | no | no | no | <64 |
| R.16 |  |  | 13 *^c^* | no | no | no | <64 |
| R.17 | Infested with F_1_ adults (GN) | 0/30 (0) | 12 *^c^* | no | no | no | <64 |
| R.18 |  |  | 12 *^c^* | no | no | no | <64 |

G.P.: guinea pig; R.: rabbit; dpi: days post inoculation or infestation.

*^a^* If guinea pig died, its blood was collected at the death day in order to be tested by serology (indirect imunofluorescence assay with *R. rickettsii* antigens); if the guinea pig did not die, its blood was collected at 21 dpi.

*^b^* Refers to number of tick pools.

*^c^* Refers to the number of engorged females (it does not include male ticks that fed with females).

*^d^* The lung of this animal was collected at necropsy and was shown to contain rickettsial DNA by PCR.

**Table S4.** Results of infestations with the second generation (F_2_) of an *Amblyomma sculptum* tick colony from Piracicaba Municipality (E-PIC), which was divided into two groups: GL+N (dually exposed to *Rickettsia rickettsii*-inoculated guinea pigs, firstly during the larval stage, secondly during the nymphal stage), and GC (uninfected control group, never exposed to infected hosts).

| **Host code** | **Experimental condition**  **(tick group)** | **No. PCR-positive ticks / No. unfed ticks tested (%)** | **No. ticks that engorged** | **Fever period (dpi)** | **Scrotal reactions (dpi)** | **Death (dpi)** | **Serological titer to *R. rickettsii ^a^*** |
| --- | --- | --- | --- | --- | --- | --- | --- |
| G.P.46 | Infested with F_2_ larvae (GC) | 0/24 (0) *^b^* | 1,201 | no | no | no | <64 |
| G.P.47 |  |  | 1,012 | no | no | no | <64 |
| G.P.48 |  |  | 1,290 | no | no | no | <64 |
| G.P.49 | Inoculated with *R. rickettsii* and infested with F_2_ larvae (GL+N) | 0/24 (0) *^b^* | 1,184 | 4 - 8 | 6 | 9 *^d^* | 128 |
| G.P.50 |  |  | 976 | 5 - 9 | 7 | 12 *^d^* | not done |
| G.P.51 |  |  | 1,079 | 6 - 8 | 7 | 11 *^d^* | not done |
| G.P.52 | Infested with F_2_ nymphs (GC) | 0/5 (0) | 91 | no | no | no | <64 |
| G.P.53 |  |  | 95 | no | no | no | <64 |
| G.P.54 |  |  | 94 | no | no | no | <64 |
| G.P.55 | Inoculated with *R. rickettsii* and infested with F_2_ nymphs (GL+N) | 3/30 (10) | 96 | 7 - 16 | 8 | no | 32,768 |
| G.P.56 |  |  | 92 | 4 - 10 | 8 | 11 *^d^* | 512 |
| G.P.57 |  |  | 81 | 4 - 8 | 6 | 10 *^d^* | 1,024 |
| R.19 | Infested with F_2_ adults (CG) | 0/10 (0) | 10 *^c^* | no | no | no | <64 |
| R.20 |  |  | 0 *^c^* | no | no | 6 *^e^* | not done |
| R.21 |  |  | 10 *^c^* | no | no | no | <64 |
| R.22 | Infested with F_2_ adults (GL+N) | 0/30 (0) | 10 *^c^* | no | no | no | <64 |
| R.23 |  |  | 8 *^c^* | no | no | no | <64 |
| R.24 |  |  | 8 *^c^* | 8 - 13 | 11 | no | 32,768 |

G.P.: guinea pig; R.: rabbit; dpi: days post inoculation or infestation.

*^a^* If guinea pig died, its blood was collected at the death day in order to be tested by serology (indirect imunofluorescence assay with *R. rickettsii* antigens); if the guinea pig did not die, its blood was collected at 21 dpi.

*^b^* Refers to number of tick pools.

*^c^* Refers to the number of engorged females (it does not include male ticks that fed with females).

*^d^* The lung of this animal was collected at necropsy and was shown to contain rickettsial DNA by PCR.

*^e^* This rabbit died of unknown cause; its lung contained no rickettsial DNA by PCR.

**Table S5.** Results of infestations with the first (F_1_) and second (F_2_) generations of an *Amblyomma sculptum* tick colony from Belo Horizonte Municipality (E-PAM), which was divided into three groups: GL (exposed to *Rickettsia rickettsii-*inoculated guinea pigs during the F_1_ larval stage), GN (exposed to *R. rickettsii-*inoculated guinea pigs during the F_1_ nymphal stage), and GC (uninfected control group, never exposed to infected hosts).

| **Host code** | **Experimental condition**  **(tick group)** | **No. PCR-positive ticks / No. unfed ticks tested (%)** | **No. ticks that engorged** | **Fever period (dpi)** | **Scrotal reactions (dpi)** | **Death (dpi)** | **Serological titer to *R. rickettsii ^a^*** |
| --- | --- | --- | --- | --- | --- | --- | --- |
| G.P.58 | Infested with F_1_ larvae (GC) | 0/19 (0) *^b^* | 1,310 | no | N.A. | no | <64 |
| G.P.59 |  |  | 1,102 | no | N.A. | no | <64 |
| G.P.60 |  |  | 1,148 | no | N.A. | no | <64 |
| G.P.61 | Inoculated with *R. rickettsii* and infested with F_1_ larvae (GL) | 0/19 (0) *^b^* | 1,096 | 4 - 7 | 5 | 9 *^d^* | <64 |
| G.P.62 |  |  | 1,175 | 4 - 7 | 5 | 9 *^d^* | 256 |
| G.P.63 |  |  | 1,245 | 3 - 8 | 4 | 10 *^d^* | 512 |
| G.P.64 | Infested with F_1_ nymphs (GC) | 0/10 (0) | 99 | no | N.A. | no | <64 |
| G.P.65 |  |  | 72 | no | N.A. | no | <64 |
| G.P.66 |  |  | 71 | no | N.A. | no | <64 |
| G.P.67 | Infested with F_1_ nymphs (GL) | 3/36 (8) | 97 | 7 - 11 | 9 | 14 | 16,384 |
| G.P.68 |  |  | 94 | 5 - 13 | 7 | no | 65,536 |
| G.P.69 |  |  | 92 | 7 - 16 | 7 | no | 32,768 |
| G.P.70 | Inoculated with *R. rickettsii* and infested with F_1_ nymphs (GN) | 0/10 (0) | 98 | 4 - 7 | N.A. | 9 *^d^* | 512 |
| G.P.71 |  |  | 99 | 4 - 7 | N.A. | 8 *^d^* | 256 |
| G.P.72 |  |  | 83 | 3 - 8 | N.A. | 9 *^d^* | 512 |
| R.25 | Infested with F_1_ adults (GC) | 0/10 (0) | 9 *^c^* | no | no | no | <64 |
| R.26 |  |  | 9 *^c^* | no | no | no | <64 |
| R.27 | Infested with F_1_ adults (GL) | 7/15 (47) | 10 *^c^* | no | no | no | <64 |
| R.28 |  |  | 12 *^c^* | 8 - 12 | 11 | no | 65,536 |
| R.29 | Infested with F_1_ adults (GN) | 10/15 (67) | 10 *^c^* | 10 - 14 | no | no | 131,072 |
| R.30 |  |  | 10 *^c^* | no | no | no | <64 |
| G.P.73 | Infested with F_2_ larvae (GL) | 10/10 (100) | 551 | 6 - 12 | 6 | no | 65,536 |
| G.P.74 | Infested with F_2_ larvae (GN) | 6/10 (100) | 264 | 5 - 11 | 6 | no | 8,192 |

G.P.: guinea pig; R.: rabbit; dpi: days post inoculation or infestation.

N.A. not applicable because the animal host was a female.

*^a^* If guinea pig died, its blood was collected at the death day in order to be tested by serology (indirect imunofluorescence assay with *R. rickettsii* antigens); if the guinea pig did not die, its blood was collected at 21 dpi.

*^b^* Refers to number of tick pools.

*^c^* Refers to the number of engorged females (it does not include male ticks that fed with females).

*^d^* The lung of this animal was collected at necropsy and was shown to contain rickettsial DNA by PCR.

**Table S6.** Results of infestations with the second generation (F_2_) of an *Amblyomma sculptum* tick colony from Belo Horizonte Municipality (E-PAM), which was divided into two groups: GL+N (dually exposed to *Rickettsia rickettsii*-inoculated guinea pigs, firstly during the larval stage, secondly during the nymphal stage), and GC (uninfected control group, never exposed to infected hosts).

| **Host code** | **Experimental condition**  **(tick group)** | **No. PCR-positive ticks / No. unfed ticks tested (%)** | **No. ticks that engorged** | **Fever period (dpi)** | **Scrotal reactions (dpi)** | **Death (dpi)** | **Serological titer to *R. rickettsii ^a^*** |
| --- | --- | --- | --- | --- | --- | --- | --- |
| G.P.75 | Infested with F_2_ larvae (GC) | 0/18 (0) *^b^* | 748 | no | N.A. | no | <64 |
| G.P.76 |  |  | 805 | no | N.A. | no | <64 |
| G.P.77 |  |  | 433 | no | N.A. | no | <64 |
| G.P.78 | Inoculated with *R. rickettsii* and infested with F_2_ larvae (GL+N) | 0/18 (0) *^b^* | 119 | no | no | 6 *^e^* | <64 |
| G.P.79 |  |  | 577 | 4 - 11 | 6 | no | 32,768 |
| G.P.80 |  |  | 682 | 4 - 11 | 6 | no | 65,536 |
| G.P.81 | Infested with F_2_ nymphs (GC) | 0/5 (0) | 26 | no | N.A. | no | <64 |
| G.P.82 |  |  | 49 | no | N.A. | no | <64 |
| G.P.83 |  |  | 70 | no | N.A. | no | <64 |
| G.P.84 | Inoculated with *R. rickettsii* and infested with F_2_ nymphs (GL+N) | 3/30 (10) | 64 | 4 - 8 | 6 | 9 *^d^* | <64 |
| G.P.85 |  |  | 63 | 5 - 9 | 6 | 11 *^d^* | 1,024 |
| G.P.86 |  |  | 41 | 4 - 13 | 6 | no | 65,536 |
| R.31 *^f^* | Infested with F_2_ adults (CG) | 0/10 (0) | 6 | no | no | no | Not done |
| R.32 *^f^* | Infested with F_2_ adults (GL+N) | 5/15 (33) | 4 | no | no | no | Not done |

G.P.: guinea pig; R.: rabbit; dpi: days post inoculation or infestation.

N.A. not applicable because the animal host was a female.

*^a^* If guinea pig died, its blood was collected at the death day in order to be tested by serology (indirect imunofluorescence assay with *R. rickettsii* antigens); if the guinea pig did not die, its blood was collected at 21 dpi.

*^b^* Refers to number of tick pools.

*^c^* Refers to the number of engorged females (it does not include male ticks that fed with females).

*^d^* The lung of this animal was collected at necropsy and was shown to contain rickettsial DNA by PCR.

*^e^* This guinea pig did not develop fever or seroconversion after rickettsial inoculation; therefore, the ticks recovered from this animal were excluded from the study.

*^f^* Due to logistic problems, only one individual rabbit could be used for infestation with adult ticks from this group.

**Table S7.** Results of infestations with the first (F_1_) and second (F_2_) generations of an *Amblyomma sculptum* tick colony from Pirassununga Municipality (NE-PIS), which was divided into three groups: GL (exposed to *Rickettsia rickettsii-*inoculated guinea pigs during the F_1_ larval stage), GN (exposed to *R. rickettsii-*inoculated guinea pigs during the F_1_ nymphal stage), and GC (uninfected control group, never exposed to infected hosts).

| **Host code** | **Experimental condition**  **(tick group)** | **No. PCR-positive ticks / No. unfed ticks tested (%)** | **No. ticks that engorged** | **Fever period (dpi)** | **Scrotal reactions (dpi)** | **Death (dpi)** | **Serological titer to *R. rickettsii ^a^*** |
| --- | --- | --- | --- | --- | --- | --- | --- |
| G.P.87 | Infested with F_1_ larvae (GC) | 0/10 (0) *^b^* | 1,108 | no | N.A. | no | <64 |
| G.P.88 |  |  | 1,135 | no | N.A. | no | <64 |
| G.P.89 |  |  | 1,188 | no | N.A. | no | <64 |
| G.P.90 | Inoculated with *R. rickettsii* and infested with F_1_ larvae (GL) | 0/10 (0) *^b^* | 1,295 | 3 - 8 | 5 | no | 16,384 |
| G.P.91 |  |  | 1,095 | 4 - 9 | 6 | no | 32,768 |
| G.P.92 |  |  | 1,176 | 4 - 9 | 6 | no | 32,768 |
| G.P.93 | Infested with F_1_ nymphs (GC) | 0/30 (0) | 68 | no | N.A. | no | <64 |
| G.P.94 |  |  | 62 | no | N.A. | no | <64 |
| G.P.95 |  |  | 74 | no | N.A. | no | <64 |
| G.P.96 | Infested with F_1_ nymphs (GL) | 1/55 (2) | 77 | 10 - 14 | 14 | no | 8,192 |
| G.P.97 |  |  | 73 | no | no | no | 512 |
| G.P.98 |  |  | 98 | no | no | no | <64 |
| G.P.99 | Inoculated with *R. rickettsii* and infested with F_1_ nymphs (GN) | 0/30 (0) | 82 | 3 - 12 | 9 | no | 16,384 |
| G.P.100 |  |  | 74 | 3 - 11 | 4 | no | 16,384 |
| G.P.101 |  |  | 54 | 3 - 9 | 4 | 10 *^d^* | not done |
| R.33 | Infested with F_1_ adults (GC) | 0/15 (0) | 14 *^c^* | no | no | no | <64 |
| R.34 |  |  | 15 *^c^* | no | no | no | <64 |
| R.35 | Infested with F_1_ adults (GL) | 0/25 (0) | 16 *^c^* | no | no | no | <64 |
| R.36 |  |  | 14 *^c^* | 9 - 13 | 12 | no | 65,536 |
| R.37 | Infested with F_1_ adults (GN) | 3/28 (11) | 15 *^c^* | 10 - 16 | 15 | no | 32,768 |
| R.38 |  |  | 15 *^c^* | 7 - 11 | 9 | no | 16,384 |
| G.P.102 | Infested with F_2_ larvae (GN) | 1/10 (10) | 272 | 5 - 10 | 7 | no | 32,768 |
| G.P.103 | Infested with F_2_ nymphs (GN) | 2/9 (22) | 87 | 6 - 11 | 8 | 13 | 2,048 |

G.P.: guinea pig; R.: rabbit; dpi: days post inoculation or infestation.

N.A. not applicable because the animal host was a female.

*^a^* If guinea pig died, its blood was collected at the death day in order to be tested by serology (indirect imunofluorescence assay with *R. rickettsii* antigens); if the guinea pig did not die, its blood was collected at 21 dpi.

*^b^* Refers to number of tick pools.

*^c^* Refers to the number of engorged females (it does not include male ticks that fed with females).

*^d^* The lung of this animal was collected at necropsy and was shown to contain rickettsial DNA by PCR.

**Table S8.** Results of infestations with the second generation (F_2_) of an *Amblyomma sculptum* tick colony from Pirassununga Municipality (NE-PIS), which was divided into two groups: GL+N (dually exposed to *Rickettsia rickettsii*-inoculated guinea pigs, firstly during the larval stage, secondly during the nymphal stage), and GC (uninfected control group, never exposed to infected hosts).

| **Host code** | **Experimental condition**  **(tick group)** | **No. PCR-positive ticks / No. unfed ticks tested (%)** | **No. ticks that engorged** | **Fever period (dpi)** | **Scrotal reactions (dpi)** | **Death (dpi)** | **Serological titer to *R. rickettsii ^a^*** |
| --- | --- | --- | --- | --- | --- | --- | --- |
| G.P.104 | Infested with F_2_ larvae (GC) | 0/29 (0) *^b^* | 1,186 | no | N.A. | no | <64 |
| G.P.105 |  |  | 1,170 | no | N.A. | no | <64 |
| G.P.106 |  |  | 1,362 | no | N.A. | no | <64 |
| G.P.107 | Inoculated with *R. rickettsii* and infested with F_2_ larvae (GL+N) | 0/29 (0) *^b^* | 1,293 | 5 - 8 | 6 | 9 *^d^* | 512 |
| G.P.108 |  |  | 1,196 | 4 - 7 | 5 | 8 *^d^* | <64 |
| G.P.109 |  |  | 1,210 | 4 - 7 | 5 | 8 *^d^* | <64 |
| G.P.110 | Infested with F_2_ nymphs (GC) *^f^* | 0/10 (0) | 89 | no | N.A. | no | <64 |
| G.P.111 |  |  | 103 | no | N.A. | no | <64 |
| G.P.113 | Inoculated with *R. rickettsii* and infested with F_2_ nymphs (GL+N) | 0/10 (0) | 73 | 4 - 7 | 5 | 9 *^d^* | <64 |
| G.P.114 |  |  | 89 | 4 - 7 | 6 | 10 *^d^* | 512 |
| G.P.115 |  |  | 50 | 4 - 7 | 6 | 9 *^d^* | 1,024 |
| R.39 | Infested with F_2_ adults (CG) | 0/9 (0) | 0 *^c^* | no | no | 5 *^e^* | <64 |
| R.40 |  |  | 9 *^c^* | no | N.A. | no | <64 |
| R.41 |  |  | 10 *^c^* | no | N.A. | no | <64 |
| R.42 | Infested with F_2_ adults (GL+N) | 0/25 (0) | 8 *^c^* | 8 - 13 | 10 | no | 32,768 |
| R.43 |  |  | 10 *^c^* | no | no | no | 32,768 |
| R.44 |  |  | 10 *^c^* | 7 - 15 | 8 | no | 8,192 |

G.P.: guinea pig; R.: rabbit; dpi: days post inoculation or infestation.

N.A. not applicable because the animal host was a female.

*^a^* If guinea pig died, its blood was collected at the death day in order to be tested by serology (indirect imunofluorescence assay with *R. rickettsii* antigens); if the guinea pig did not die, its blood was collected at 21 dpi.

*^b^* Refers to number of tick pools.

*^c^* Refers to the number of engorged females (it does not include male ticks that fed with females).

*^d^* The lung of this animal was collected at necropsy and was shown to contain rickettsial DNA by PCR.

*^e^* This rabbit died of unknown cause; its lung contained no rickettsial DNA by PCR.

*^f^* Due to logistic reasons, only two guinea pigs could be used for this nymphal infestation group.

**Table S9.** Results of infestations with the first generation (F_1_) of an *Amblyomma sculptum* tick colony from Poconé Municipality (NE-POC), which was divided into three groups: GL (exposed to *Rickettsia rickettsii-*inoculated guinea pigs during the larval stage), GN (exposed to *R. rickettsii-*inoculated guinea pigs during the nymphal stage), and GC (uninfected control group, never exposed to infected hosts).

| **Host code** | **Experimental condition**  **(tick group)** | **No. PCR-positive ticks / No. unfed ticks tested (%)** | **No. ticks that engorged** | **Fever period (dpi)** | **Scrotal reactions (dpi)** | **Death (dpi)** | **Serological titer to *R. rickettsii ^a^*** |
| --- | --- | --- | --- | --- | --- | --- | --- |
| G.P.116 | Infested with F_1_ larvae (GC) | 0/10 (0) *^b^* | 1,304 | no | N.A. | no | <64 |
| G.P.117 |  |  | 934 | no | N.A. | no | <64 |
| G.P.118 |  |  | 1,422 | no | N.A. | no | <64 |
| G.P.119 | Inoculated with *R. rickettsii* and infested with F_1_ larvae (GL) | 0/10 (0) *^b^* | 900 | 4 - 8 | 6 | 10 *^d^* | not done |
| G.P.120 |  |  | 1,116 | 4 - 7 | 5 | 9 *^d^* | not done |
| G.P.121 |  |  | 1,459 | 4 - 8 | 6 | 9 *^d^* | not done |
| G.P.122 | Infested with F_1_ nymphs (GC) | 0/10 (0) | 62 | no | N.A. | no | <64 |
| G.P.123 |  |  | 75 | no | N.A. | no | <64 |
| G.P.124 |  |  | 70 | no | N.A. | no | <64 |
| G.P.125 | Infested with F_1_ nymphs (GL) | 1/80 (1) | 71 | no | no | no | <64 |
| G.P.126 |  |  | 69 | no | no | no | <64 |
| G.P.127 |  |  | 64 | no | no | no | <64 |
| G.P.128 | Inoculated with *R. rickettsii* and infested with F_1_ nymphs (GN) | 0/10 (0) | 40 | 3 - 6 | 5 | 8 *^d^* | not done |
| G.P.129 |  |  | 62 | 3 - 7 | N.A. | 9 *^d^* | not done |
| G.P.130 |  |  | 69 | 3 - 6 | N.A. | 8 *^d^* | not done |
| R.45 | Infested with F_1_ adults (GC) | 0/10 (0) | 11 *^c^* | no | no | no | <64 |
| R.46 |  |  | 9 *^c^* | no | no | no | <64 |
| R.47 | Infested with F_1_ adults (GL) | 0/20 (0) | 20 *^c^* | no | no | no | <64 |
| R.48 |  |  | 17 *^c^* | no | no | no | <64 |
| R.49 | Infested with F_1_ adults (GN) | 0/15 (0) | 8 *^c^* | no | no | no | <64 |
| R.50 |  |  | 7 *^c^* | no | no | no | <64 |

G.P.: guinea pig; R.: rabbit; dpi: days post inoculation or infestation.

N.A. not applicable because the animal host was a female.

*^a^* If guinea pig died, its blood was collected at the death day in order to be tested by serology (indirect imunofluorescence assay with *R. rickettsii* antigens); if the guinea pig did not die, its blood was collected at 21 dpi.

*^b^* Refers to number of tick pools.

*^c^* Refers to the number of engorged females (it does not include male ticks that fed with females).

*^d^* The lung of this animal was collected at necropsy and was shown to contain rickettsial DNA by PCR.

**Table S10.** Results of infestations with the second generation (F_2_) of an *Amblyomma sculptum* tick colony from Poconé Municipality (NE-POC), which was divided into two groups: GL+N (dually exposed to *Rickettsia rickettsii*-inoculated guinea pigs, firstly during the larval stage, secondly during the nymphal stage), and GC (uninfected control group, never exposed to infected hosts).

| **Host code** | **Experimental condition**  **(tick group)** | **No. PCR-positive ticks / No. unfed ticks tested (%)** | **No. ticks that engorged** | **Fever period (dpi)** | **Scrotal reactions (dpi)** | **Death (dpi)** | **Serological titer to *R. rickettsii ^a^*** |
| --- | --- | --- | --- | --- | --- | --- | --- |
| G.P.131 | Infested with F_2_ larvae (GC) | 0/20 (0) *^b^* | 899 | no | N.A. | no | <64 |
| G.P.132 |  |  | 902 | no | N.A. | no | <64 |
| G.P.133 |  |  | 872 | no | N.A. | no | <64 |
| G.P.134 | Inoculated with *R. rickettsii* and infested with F_2_ larvae (GL+N) | 0/20 (0) *^b^* | 901 | 4 - 11 | 7 | no | 65,536 |
| G.P.135 |  |  | 1,030 | 4 - 8 | 7 | 9 *^d^* | 512 |
| G.P.136 |  |  | 1,711 | 4 - 11 | 7 | no | 32,768 |
| G.P.137 | Infested with F_2_ nymphs (GC) *^f^* | 0/10 (0) | 81 | no | N.A. | no | <64 |
| G.P.138 |  |  | 91 | no | N.A. | no | <64 |
| G.P.139 |  |  | 88 | no | N.A. | no | <64 |
| G.P.140 | Inoculated with *R. rickettsii* and infested with F_2_ nymphs (GL+N) | 0/15 (0) | 69 | 3 - 13 | 4 | no | 32,768 |
| G.P.141 |  |  | 99 | 3 - 7 | 4 | 8 *^d^* | 256 |
| G.P.142 |  |  | 77 | 3 - 8 | 4 | 9 *^d^* | 512 |
| R.51 | Infested with F_2_ adults (CG) | 0/10 (0) | 6 *^c^* | no | no | no | <64 |
| R.52 |  |  | 10 *^c^* | no | no | no | <64 |
| R.53 |  |  | 9 *^c^* | no | no | no | <64 |
| R.54 | Infested with F_2_ adults (GL+N) | 3/15 (20) | 6 *^c^* | 10 - 15 | 14 | no | 32,768 |
| R.55 |  |  | 6 *^c^* | 7 - 12 | 12 | no | 32,768 |
| R.56 |  |  | 8 *^c^* | 7 - 12 | no | no | 131,072 |

G.P.: guinea pig; R.: rabbit; dpi: days post inoculation or infestation.

N.A. not applicable because the animal host was a female.

*^a^* If guinea pig died, its blood was collected at the death day in order to be tested by serology (indirect imunofluorescence assay with *R. rickettsii* antigens); if the guinea pig did not die, its blood was collected at 21 dpi.

*^b^* Refers to number of tick pools.

*^c^* Refers to the number of engorged females (it does not include male ticks that fed with females).

*^d^* The lung of this animal was collected at necropsy and was shown to contain rickettsial DNA by PCR.

**Table S11.** Results of infestations with the first generation (F_1_) of an *Amblyomma sculptum* tick colony from Chapada Gaúcha Municipality (NE-GSV), which was divided into three groups: GL (exposed to *Rickettsia rickettsii-*inoculated guinea pigs during the larval stage), GN (exposed to *R. rickettsii-*inoculated guinea pigs during the nymphal stage), and GC (uninfected control group, never exposed to infected hosts).

| **Host code** | **Experimental condition**  **(tick group)** | **No. PCR-positive ticks / No. unfed ticks tested (%)** | **No. ticks that engorged** | **Fever period (dpi)** | **Scrotal reactions (dpi)** | **Death (dpi)** | **Serological titer to *R. rickettsii ^a^*** |
| --- | --- | --- | --- | --- | --- | --- | --- |
| G.P.143 | Infested with F_1_ larvae (GC) | 0/20 (0) *^b^* | 1,824 | no | N.A. | no | <64 |
| G.P.144 |  |  | 1,756 | no | N.A. | no | <64 |
| G.P.145 |  |  | 2,025 | no | N.A. | no | <64 |
| G.P.146 | Inoculated with *R. rickettsii* and infested with F_1_ larvae (GL) | 0/20 (0) *^b^* | 1,403 | 4 - 6 | 6 | 8 *^d^* | not done |
| G.P.147 |  |  | 2,118 | 4 - 8 | 5 | 10 *^d^* | not done |
| G.P.148 |  |  | 1,786 | 3 - 6 | 5 | 9 *^d^* | not done |
| G.P.150 | Infested with F_1_ nymphs (GC) | 0/28 (0) | 72 | no | N.A. | no | <64 |
| G.P.151 |  |  | 59 | no | N.A. | no | <64 |
| G.P.152 |  |  | 52 | no | N.A. | no | <64 |
| G.P.153 | Infested with F_1_ nymphs (GL) | 0/48 (0) | 99 | 8 - 12 | 9 | no | 4,096 |
| G.P.154 |  |  | 97 | no | no | no | <64 |
| G.P.155 |  |  | 82 | no | no | no | <64 |
| G.P.156 | Inoculated with *R. rickettsii* and infested with F_1_ nymphs (GN) | 0/28 (0) | 78 | 4 - 7 | 5 | 10 *^d^* | 256 |
| G.P.157 |  |  | 77 | 4 - 7 | 6 | 10 *^d^* | 512 |
| G.P.158 |  |  | 67 | 4 - 6 | 6 | 8 *^d^* | 128 |
| R.57 | Infested with F_1_ adults (GC) | 0/15 (0) | 14 *^c^* | no | no | no | <64 |
| R.58 |  |  | 15 *^c^* | no | no | no | <64 |
| R.59 | Infested with F_1_ adults (GL) | 0/16 (0) | 14 *^c^* | no | no | no | <64 |
| R.60 |  |  | 14 *^c^* | 9 - 13 | 12 | no | 8,192 |
| R.61 | Infested with F_1_ adults (GN) | 4/22 (18) | 15 *^c^* | no | no | no | <64 |
| R.62 |  |  | 14 *^c^* | 6 - 12 | no | no | 16,384 |

G.P.: guinea pig; R.: rabbit; dpi: days post inoculation or infestation.

N.A. not applicable because the animal host was a female.

*^a^* If guinea pig died, its blood was collected at the death day in order to be tested by serology (indirect imunofluorescence assay with *R. rickettsii* antigens); if the guinea pig did not die, its blood was collected at 21 dpi.

*^b^* Refers to number of tick pools.

*^c^* Refers to the number of engorged females (it does not include male ticks that fed with females).

*^d^* The lung of this animal was collected at necropsy and was shown to contain rickettsial DNA by PCR.

**Table S12.** Results of infestations with the second (F_2_) and third (F_3_) generations of an *Amblyomma sculptum* tick colony from Chapada Gaúcha Municipality (NE-GSV), which was divided into two groups: GL+N (dually exposed to *Rickettsia rickettsii*-inoculated guinea pigs, firstly during the F_2_ larval stage, secondly during the F_2_ nymphal stage), and GC (uninfected control group, never exposed to infected hosts).

| **Host code** | **Experimental condition**  **(tick group)** | **No. PCR-positive ticks / No. unfed ticks tested (%)** | **No. ticks that engorged** | **Fever period (dpi)** | **Scrotal reactions (dpi)** | **Death (dpi)** | **Serological titer to *R. rickettsii ^a^*** |
| --- | --- | --- | --- | --- | --- | --- | --- |
| G.P.159 | Infested with F_2_ larvae (GC) | 0/29 (0) *^b^* | 880 | no | N.A. | no | <64 |
| G.P.160 |  |  | 995 | no | N.A. | no | <64 |
| G.P.161 |  |  | 1,094 | no | N.A. | no | <64 |
| G.P.162 | Inoculated with *R. rickettsii* and infested with F_2_ larvae (GL+N) | 0/29 (0) *^b^* | 1,018 | 3 - 6 | 6 | 10 *^d^* | 512 |
| G.P.163 |  |  | 753 | 4 - 9 | 8 | 11 *^d^* | 16,384 |
| G.P.164 |  |  | 822 | 4 - 6 | 6 | 8 *^d^* | 256 |
| G.P.165 | Infested with F_2_ nymphs (GC) *^f^* | 0/10 (0) | 53 | no | N.A. | no | <64 |
| G.P.166 |  |  | 97 | no | N.A. | no | <64 |
| G.P.167 |  |  | 66 | no | N.A. | no | <64 |
| G.P.168 | Inoculated with *R. rickettsii* and infested with F_2_ nymphs (GL+N) | 5/31 (16) | 94 | 3 - 6 | 4 | 8 *^d^* | 256 |
| G.P.169 |  |  | 54 | 3 - 6 | 4 | 8 *^d^* | <64 |
| G.P.170 |  |  | 77 | 4 - 7 | 6 | 10 *^d^* | 512 |
| R.63 | Infested with F_2_ adults (CG) | 0/10 (0) | 6 *^c^* | no | no | no | <64 |
| R.52 |  |  | 9 *^c^* | no | no | no | <64 |
| R.53 |  |  | 10 *^c^* | no | no | no | <64 |
| R.54 | Infested with F_2_ adults (GL+N) | 7/30 (23) | 11 *^c^* | no | no | no | <64 |
| R.55 |  |  | 10 *^c^* | 6 - 9 | 11 | no | 16,384 |
| R.56 |  |  | 11 *^c^* | 6 - 10 | 11 | 13 | 32,768 |
| G.P.171 | Infested with F_3_ larvae (GL+N) | 9/10 (90) | 520 | 6 - 10 | 8 | no | 16,834 |
| G.P.172 |  | 9/10 (90) | 75 | 7 - 12 | 8 | no | 4,096 |
| G.P.173 |  | 3/10 (30) | 2 | no | no | no | 8,192 |

G.P.: guinea pig; R.: rabbit; dpi: days post inoculation or infestation.

N.A. not applicable because the animal host was a female.

*^a^* If guinea pig died, its blood was collected at the death day in order to be tested by serology (indirect imunofluorescence assay with *R. rickettsii* antigens); if the guinea pig did not die, its blood was collected at 21 dpi.

*^b^* Refers to number of tick pools.

*^c^* Refers to the number of engorged females (it does not include male ticks that fed with females).

*^d^* The lung of this animal was collected at necropsy and was shown to contain rickettsial DNA by PCR.

**Table S13**. Filial infection rates (FIR: No. infected eggs or larvae / No. tested eggs or larvae x 100) for the offspring of *Rickettsia rickettsii-*infected engorged females of five tick colonies of *Amblyomma sculptum*.

| Tick colony | Infected females with rickettsial transovarial transmission *^a^* | No. infected eggs / No. tested eggs (FIR) | No. infected larvae / No. tested larvae (FIR) |
| --- | --- | --- | --- |
| E-ITU | GL female 1 | 10/10 (100) | 10/10 (100) |
|  | GL female 2 | 10/10 (100) | 10/10 (100) |
|  | GN female 1 | 10/10 (100) | Not tested |
|  | GN female 2 | 9/10 (90) | Not tested |
|  | GN female 3 | 10/10 (100) | Not tested |
|  | GL+N female 1 | 10/10 (100) | 10/10 (100) |
|  | GL+N female 2 | 10/10 (100) | 10/10 (100) |
|  | GL+N female 3 | 10/10 (100) | Not tested |
|  | GL+N female 4 | 0/10 (0) | 0/10 (0) |
| E-PAM | GL female 1 | 10/10 (100) | 10/10 (100) |
|  | GN female 1 | 6/10 (60 | 6/10 (60 |
| NE-PIS | GL female 1 | 1/10 (10) | Not tested |
|  | GL female 2 | 0/10 (0) | Not tested |
|  | GN female 1 | 5/10 (50) | 1/10 (10) |
|  | GL+N female 1 | 8/10 (80) | 5/10 (50) |
|  | GL+N female 2 | 10/10 (100) | 5/10 (50) |
|  | GL+N female 3 | 6/10 (60) | 5/10 (50) |
| NE-POC | GL+N female 1 | 10/10 (100) | Not tested |
| NE-GSV | GN female 1 | 7/10 (70) | Not tested |
|  | GL+N female 1 | 10/10 (100) | 9/10 (90) |
|  | GL+N female 2 | 6/10 (60) | 9/10 (90) |
|  | GL+N female 3 | 3/10 (30) | 3/10 (30) |

*^a^* GL and GN females consisted of ticks that were previously exposed to *R. rickettsii* acquisition feeding as larvae and nymphs, respectively, which fed on rickettsemic guinea pigs; GL+N were dually exposed (as larvae and nymphs) to rickettsemic guinea pigs.

**Table S14.** Biological and reproductive parameters of engorged females of an *Amblyomma sculptum* tick colony from Itu Municipality (E-ITU), which was divided into four tick groups: GL, GN, GL+GN, and CG. In each group, engorged females were divided into two subgroups (infected by *Rickettsia ricketsii* or non-infected), as determined by testing the engorged female by PCR at the end of the oviposition period.

| **Biological parameter** | **Tick groups *^a^*** | | | | | | | | | |
| --- | --- | --- | --- | --- | --- | --- | --- | --- | --- | --- |
|  | **GL** | |  | **GN** | |  | **GL+N** | |  | **GC *^c^*** |
|  | **Infected** | **Non-infected** |  | **Infected** | **Non-infected** |  | **Infected** | **Non-infected** |  | **Non-infected** |
| No. recovered engorged females | 16 | 6 |  | 11 | 10 |  | 20 | 2 |  | 33 |
| Engorged female weight (mg) *^b,c^* | 316.2 ± 100.5  (154.3-501.9)a | 406.5 ± 192.7  (165.5-649.4)a,b,c |  | 378.4 ± 154.4  (142.5-592.0)a,c | 508.0 ± 90.7  (405.9-654.7)b |  | 363.2 ± 97.2  (173.7-553.9)a | 460.5 ± 98.5  (390.8-530.1)a,b,c |  | 422.7 ± 112.1  (241.8 – 672.8)c |
| Feeding period (days) *^b^* | 12.4 ± 2.6 (9-19) | 12.8 ± 3.0 (9-19) |  | 11.4 ± 1.2 (9-13) | 11.8 ± 1.0 (10-13) |  | 12.0 ± 2.5 (10-17) | 13.5 ± 2.1 (12-15) |  | 10.5 ± 1.7 (8-16) |
| No. females that oviposited (%) | 15(94) | 6 (100) |  | 10 (91) | 10 (100) |  | 20 (100) | 2 (100) |  | 32 (97) |
| Preoviposition period (days) *^b^* | 7.9 ± 1.4 (5-10) | 7.5 ± 2.4 (4-10) |  | 9.0 ± 4.6 (5-20) | 8.5 ± 4.6 (5-21) |  | 6.8 ± 1.2 (6-11) | 5.5 ± 0.7 (5-6) |  | 7.1 ± 1.0 (5-9) |
| Egg mass weight (mg) *^b,c^* | 106.6 ± 65.6  (20.4-231.5)a,d | 125.7 ± 96.1  (29.1-232.4)a,c,d |  | 114.0 ± 73.2  (0.7-200.1)a,b | 173.4 ± 75.5  (0.9–278.5)b.e |  | 164.0 ± 69.9  (12.3–283.0)b,c,e | 183.3 ± 72.2  (132.2-234.3)a,d,e |  | 191.2 ± 81.5  (16.4-347.7)d,e |
| CEI *^b,c^* | 31.4 ± 12.9  (7.3 - 47.5)a | 27.4 ± 12.5  (14.2 - 45.1)a |  | 29.2 ± 17.3  (0.2 - 60.2)a | 34.3 ± 14.3  (0.2 - 49.5)a,b |  | 43.3 ± 12.8  (7.1 - 55.6)b,d | 39.0 ± 7.3  (33.8 - 44.2)a,d |  | 44.2 ± 12.3  (5.7 - 56.5)d |
| Egg incubation period (days) *^b^* | 36.8 ± 2.3 (33-42) | 36.8 ± 1.7 (35-39) |  | 36.8 ± 1.5 (35-39) | 35.6 ± 1.4 (33-38) |  | 35.9 ± 1.6 (33-39) | 44.0 ± 0.0 (44) |  | 35.1 ± 1.2 (33-38) |
| No. females with fertile eggs (%) *^c^* | 12/15 (80)a | 4/6 (67)a |  | 6/10 (60)a | 9/10 (90)a |  | 14/20 (70)a | 1/2 (50)a |  | 25/32 (78)a |
| % egg hatching *^b,c^* | 16.4 ± 21.7 (0-60)a | 30.2 ± 35.0 (0-80)a,b,c |  | 16.5 ± 23.3 (0-80)a | 62.0 ± 30.1 (60-90)b,d |  | 23.1 ± 30.8 (0-95)a | 0.05 ± 0.07 (0-0.1)c |  | 83.0 ± 14.9 (40-99)d |

*^a^* GL: ticks exposed to *Rickettsia rickettsii-*inoculated guinea pigs during the larval stage; GN: ticks exposed to *R. rickettsii-*inoculated guinea pigs during the nymphal stage; GL+N: ticks exposed to *R. rickettsii-*inoculated guinea pigs during both the larval and nymphal stages; GC: uninfected control group, never exposed to infected hosts.

*^b^* Values presented as: mean ± standard deviation (range).

CEI: conversion efficiency index = egg mass weight/ engorged female weight × 100.

*^c^* includes females of both F_1_ and F_2_ GC groups

d Different letters in the same line mean significantly different values (*P*<0.05).

**Table S15.** Biological and reproductive parameters of engorged females of an *Amblyomma sculptum* tick colony from Piracicaba Municipality (E-PIC), which was divided into four tick groups: GL, GN, GL+GN, and CG. In each group, engorged females were divided into two subgroups (infected by *Rickettsia ricketsii* or non-infected), as determined by testing the engorged female by PCR at the end of the oviposition period.

| **Biological parameter** | **Tick groups *^a^*** | | | | | | | | | |
| --- | --- | --- | --- | --- | --- | --- | --- | --- | --- | --- |
|  | **GL** | |  | **GN** | |  | **GL+N** | |  | **GC** |
|  | **Infected** | **Non-infected** |  | **Infected** | **Non-infected** |  | **Infected** | **Non-infected** |  | **Non-infected** |
| No. recovered engorged females | 0 | 23 |  | 0 | 24 |  | 2 | 24 |  | 42 |
| Engorged female weight (mg) *^b,c^* |  | 778.4 ± 207.7  (334.6-1243.0)a |  |  | 896.8 ± 161.3  (517.6-1135.0)b |  | 512.6 ± 1.77  (511.3-513.8)c | 672.0 ± 303.9  (99.8-1507.0)a |  | 819.5 ± 273.7  (133.9-1237.0)a,b |
| Feeding period (days) *^b^* |  | 8.5 ± 1.1 (7-10) |  |  | 9.4 ± 1.5 (7-12) |  | 11.0 ± 2.8 (9-13) | 9.4 ± 1.4 (8-13) |  | 9.2 ± 1.5 (7-13) |
| No. females that oviposited (%) |  | 22 (96) |  |  | 24 (100) |  | 2 (100) | 24 (100) |  | 42 (100) |
| Preoviposition period (days) *^b^* |  | 6.9 ± 1.1 (5-10) |  |  | 6.8 ± 1.3 (4-10) |  | 6.5 ± 2.1 (5-8) | 6.5 ± 1.5 (5-12) |  | 5.8 ± 1.1 (3-8) |
| Egg mass weight (mg) *^b,c^* |  | 392.5 ± 140.7  (36.0-704.1)a,c |  |  | 423.2 ± 118.6  (141.0-596.3)a |  | 192.0 ± 17.9  (179.3-204.6)b | 302.3 ± 173.3  (66.4-793.5)c |  | 423.2 ± 181.9  (71.6-689.7)a |
| CEI *^b,c^* |  | 47.6 ± 10.3  (9.3-57.6)a |  |  | 47.0 ± 9.2  (13.4-55.2)a |  | 37.4 ± 3.4  (35.1-39.8)a,b | 41.0 ± 9.8  (12.7-56.2)b |  | 47.6 ± 11.2  (17.6-63.8)a |
| Egg incubation period (days) *^b^* |  | 34.7 ± 1.1 (33-37) |  |  | 34.7 ± 1.4 (33-38) |  | 34.5 ± 0.7 (34-35) | 35.6 ± 1.1 (34-37) |  | 35.4 ± 1.5 (32-39) |
| No. females with fertile eggs (%) *^c^* |  | 19/22 (86)a |  |  | 23/24 (96)a |  | 1/2 (50)a | 20/24 (83)a |  | 39/42 (93)a |
| % egg hatching *^b,c^* |  | 52.1 ± 35.8 (0-99)a |  |  | 66.3 ± 25.3 (0-99)a,b |  | 65.0 ± 21.2 (50-80)a,b | 63.1 ± 33.7 (0-95)a,b |  | 76.4 ± 30.1 (0-99)b |

*^a^* GL: ticks exposed to *Rickettsia rickettsii-*inoculated guinea pigs during the larval stage; GN: ticks exposed to *R. rickettsii-*inoculated guinea pigs during the nymphal stage; GL+N: ticks exposed to *R. rickettsii-*inoculated guinea pigs during both the larval and nymphal stages; GC: uninfected control group, never exposed to infected hosts.

*^b^* Values presented as: mean ± standard deviation (range).

CEI: conversion efficiency index = egg mass weight/ engorged female weight × 100.

*^c^* Different letters in the same line mean significantly different values (*P*<0.05).

**Table S16.** Biological and reproductive parameters of engorged females of an *Amblyomma sculptum* tick colony from Belo Horizonte Municipality (E-PAM), which was divided into four tick groups: GL, GN, GL+GN, and CG. In each group, engorged females were divided into two subgroups (infected by *Rickettsia ricketsii* or non-infected), as determined by testing the engorged female by PCR at the end of the oviposition period.

| **Biological parameter** | **Tick groups *^a^*** | | | | | | | | | |
| --- | --- | --- | --- | --- | --- | --- | --- | --- | --- | --- |
|  | **GL** | |  | **GN** | |  | **GL+N** | |  | **GC** |
|  | **Infected** | **Non-infected** |  | **Infected** | **Non-infected** |  | **Infected** | **Non-infected** |  | **Non-infected** |
| No. recovered engorged females | 7 | 15 |  | 6 | 14 |  | 0 | 4 |  | 24 |
| Engorged female weight (mg) *^b,c^* | 472.4 ± 190.6  (198.5-758.2)a | 518.7 ± 186.7  (134.5-824.9)a |  | 578.6 ± 173.8  (436.6-880.7)a,b | 699.0 ± 153.4  (315.5-937.3)b |  |  | 137.4 ± 27.7  (100.8-166.4)c |  | 554.7 ± 225.7  (221.0-1012.0)a |
| Feeding period (days) *^b^* | 12.1 ± 2.9 (9-17) | 10.7 ± 2.1 (9-16) |  | 8.7 ± 1.2 (7-10) | 8.4 ± 1.2 (7-11) |  |  | 13.3 ± 3.2 (10-16) |  | 9.8 ± 1.2 (8-13) |
| No. females that oviposited (%) | 7 (100) | 15 (100) |  | 6 (100) | 14 (100) |  |  | 4 (100) |  | 24 (100) |
| Preoviposition period (days) *^b^* | 7.9 ± 1.3 (5-9) | 7.2 ± 1.4 (5-10) |  | 6.3 ± 0.5 (6-7) | 8.1 ± 2.8 (6-16) |  |  | 8.3 ± 1.5 (6-9) |  | 7.8 ± 1.9 (6-14) |
| Egg mass weight (mg) *^b,c^* | 161.5 ± 151.9  (18.0-433.6)a,c | 295.6 ± 113.3  (42.3-497.5)a,b |  | 279.2 ± 147.9  (134.9-508.5)a,b | 321.2 ± 139.3  (89.7-564.9)b |  |  | 45.7 ± 10.7  (30.2-53.0)c |  | 246.0 ± 127.8  (42.5-583.4)a,b |
| CEI *^b,c^* | 32.2 ± 23.2  (5.7-57.2)a,c | 56.6 ± 11.6  (31.5-69.3)b |  | 46.4 ± 13.2  (26.7-59.3)a,b,c | 45.7 ± 14.7  (10.8-60.3)a |  |  | 33.1 ± 2.7  (29.9-35.7)c |  | 42.8 ± 12.3  (19.2-58.5)a |
| Egg incubation period (days) *^b^* | 38.3 ± 1.0 (37-40) | 38.2 ± 0.9 (37-40) |  | 38.5 ± 1.0 (37-40) | 38.4 ± 0.9 (34-40) |  |  |  |  | 38.2 ± 1.0 (37-40) |
| No. females with fertile eggs (%) *^c^* | 6/7 (86)a | 15/15 (100)a |  | 6/6 (100)a | 12/14 (86)a |  |  | 0/4 (0) |  | 18/24 (75)a |
| % egg hatching *^b,c^* | 75.7 ± 38.2 (0-99)a | 72.4 ± 35.9 (1-99)a |  | 55.0 ± 27.4 (20-80)a | 69.9 ± 40.3 (0-99)a |  |  | 0 ± 0 (0-0) |  | 59.5 ± 47.6 (0-99)a |

*^a^* GL: ticks exposed to *Rickettsia rickettsii-*inoculated guinea pigs during the larval stage; GN: ticks exposed to *R. rickettsii-*inoculated guinea pigs during the nymphal stage; GL+N: ticks exposed to *R. rickettsii-*inoculated guinea pigs during both the larval and nymphal stages; GC: uninfected control group, never exposed to infected hosts.

*^b^* Values presented as: mean ± standard deviation (range).

CEI: conversion efficiency index = egg mass weight/ engorged female weight × 100.

*^c^* Different letters in the same line mean significantly different values (*P*<0.05).

**Table S17.** Biological and reproductive parameters of engorged females of an *Amblyomma sculptum* tick colony from Pirassununga Municipality (NE-PIS), which was divided into four tick groups: GL, GN, GL+GN, and CG. In each group, engorged females were divided into two subgroups (infected by *Rickettsia ricketsii* or non-infected), as determined by testing the engorged female by PCR at the end of the oviposition period.

| **Biological parameter** | **Tick groups *^a^*** | | | | | | | | | |
| --- | --- | --- | --- | --- | --- | --- | --- | --- | --- | --- |
|  | **GL** | |  | **GN** | |  | **GL+N** | |  | **GC** |
|  | **Infected** | **Non-infected** |  | **Infected** | **Non-infected** |  | **Infected** | **Non-infected** |  | **Non-infected** |
| No. recovered engorged females | 11 | 19 |  | 15 | 15 |  | 19 | 9 |  | 48 |
| Engorged female weight (mg) *^b,c^* | 660.7 ± 363.8  (110.9-1269.0)a,b,c | 558.8 ± 284.6  (208.1-1002.0)b |  | 834.8 ± 124.2  (657.5-1009.0)a,c | 881.9 ± 193.5  (354.5-1156.0)c |  | 811.0 ± 237.5  (320.6-1254.0)a,c | 709.4 ± 161.8  (451.0-1031.0)a,b |  | 826.0 ± 182.2  (242.0-1113.0)a,c |
| Feeding period (days) *^b^* | 10.7 ± 1.9 (8-14) | 10.2 ± 1.7 (8-14) |  | 8.9 ± 1.4 (7-12) | 9.5 ± 1.5 (7-12) |  | 8.5 ± 0.5 (8-9) | 8.4 ± 0.9 (7-10) |  | 8.8 ± 1.2 (7-13) |
| No. females that oviposited (%) | 11 (100) | 19 (100) |  | 14 (93) | 15 (100) |  | 18 (95) | 9 (100) |  | 45 (94) |
| Preoviposition period (days) *^b^* | 6.4 ± 1.7 (3-10) | 6.4 ± 1.3 (3-9) |  | 6.7 ± 1.1 (4-8) | 6.0 ± 1.7 (4-11) |  | 6.5 ± 0.8 (5-8) | 6.2 ± 0.8 (5-8) |  | 6.7 ± 1.7 (5-15) |
| Egg mass weight (mg) *^b,c^* | 259.0 ± 202.3  (15.9-677.7)a | 259.7 ± 162.3  (81.3-583.1)a |  | 416.1 ± 124.0  (109.5-569.0)b | 436.4 ± 125.9  (111.7-617.7)b |  | 417.0 ± 154.2  (76.8-631.0)b | 362.9 ± 124.2  (189.1-590.9)a,b |  | 433.4 ± 142.9  (2.2-639.9)b |
| CEI *^b,c^* | 35.4 ± 15.5  (12.7-53.4)a | 46.6 ± 14.0  (11.6-62.7)a,b |  | 48.4 ± 11.5  (16.3-59.7)b | 48.5 ± 6.5  (31.5-55.5)b |  | 51.5 ± 13.5  (23.7-74.7)b | 50.8 ±10.7  (27.7-61.2)b |  | 51.3 ± 12.5  (0.3-63.9)b |
| Egg incubation period (days) *^b^* | 39.5 ± 2.3 (36-45) | 40.1 ± 1.7 (36-43) |  | 40.2 ± 1.2 (39-43) | 40.2 ± 1.5 (37-42) |  | 38.6 ±1.6 (35-42) | 38.3 ± 1.3 (36-40) |  | 39.4 ± 1.8 (36-45) |
| No. females with fertile eggs (%) *^c^* | 7/11 (64)a | 17/19 (89)a,b |  | 13/14 (93)a,b | 13/15 (87)a,b |  | 17/18 (94)a,b | 9/9 (100)a,b |  | 43/45 (96)b |
| % egg hatching *^b,c^* | 22.7 ± 26.1 (0-70)a | 60.0 ± 38.57 (0-99)b |  | 57.7 ± 39.7 (0-99)b | 58.2 ± 40.9 (0-99)b |  | 74.1 ± 22.6 (0-99)b | 78.9 ± 10.5 (60-90)b |  | 74.2 ± 30.9 (0-99)b |

*^a^* GL: ticks exposed to *Rickettsia rickettsii-*inoculated guinea pigs during the larval stage; GN: ticks exposed to *R. rickettsii-*inoculated guinea pigs during the nymphal stage; GL+N: ticks exposed to *R. rickettsii-*inoculated guinea pigs during both the larval and nymphal stages; GC: uninfected control group, never exposed to infected hosts.

*^b^* Values presented as: mean ± standard deviation (range).

CEI: conversion efficiency index = egg mass weight/ engorged female weight × 100.

*^c^* Different letters in the same line mean significantly different values (*P*<0.05).

**Table S18.** Biological and reproductive parameters of engorged females of an *Amblyomma sculptum* tick colony from Poconé Municipality (NE-POC), which was divided into four tick groups: GL, GN, GL+GN, and CG. In each group, engorged females were divided into two subgroups (infected by *Rickettsia ricketsii* or non-infected), as determined by testing the engorged female by PCR at the end of the oviposition period.

| **Biological parameter** | **Tick groups *^a^*** | | | | | | | | | |  |
| --- | --- | --- | --- | --- | --- | --- | --- | --- | --- | --- | --- |
|  | **GL** | |  | **GN** | |  | **GL+N** | |  | **GC** |  |
|  | **Infected** | **Non-infected** |  | **Infected** | **Non-infected** |  | **Infected** | **Non-infected** |  | **Non-infected** |  |
| No. recovered engorged females | 0 | 37 |  | 0 | 15 |  | 8 | 12 |  | 44 |  |
| Engorged female weight (mg) *^b,c^* |  | 648.2 ± 171  (377.3-986.6)a |  |  | 519.8 ± 197.4  (203.3-820.4)b |  | 644.1 ± 300.3  (340.3-1200.6)a,b | 720.3 ± 251.8  (137.6-1130.0)a |  | 617.0 ± 186.7  (176.2-953.2)a,b |  |
| Feeding period (days) *^b^* |  | 11.1 ± 2.1 (8-18) |  |  | 12.5 ± 3.4 (8-21) |  | 10.5 ± 2.3 (9-16) | 12.3 ± 2.3 (10-17) |  | 11.1 ± 1.8 (8-15) |  |
| No. females that oviposited (%) |  | 37 (100) |  |  | 15 (100) |  | 7 (87) | 12 (100) |  | 44 (100) |  |
| Preoviposition period (days) *^b^* |  | 6.6 ± 1.1 (5-10) |  |  | 6.0 ± 1.2 (4-8) |  | 6.7 ± 0.8 (6-8) | 6.8 ± 0.6 (6-8) |  | 6.7 ± 1.0 (4-9) |  |
| Egg mass weight (mg) *^b,c^* |  | 344.0 ± 130.5  (94.1-605.6)a |  |  | 245.8 ± 137.1  (47.4-465.5)b |  | 302.0 ± 156.2  (110.6-549.5)a,b | 250.1 ± 154.0  (41.3-562.0)a,b |  | 297.5 ± 127.0  (47.9-578.4)a,b |  |
| CEI *^b,c^* |  | 51.7 ± 10.2  (20.5-66.0)a |  |  | 44.4 ± 13.1  (16.8-59.2)a,b,c |  | 42.4 ± 5.6  (32.5-48.7)b,c | 34.5 ± 14.6  (30.0-52.5)b |  | 46.7 ± 10.5  (22.6-60.7)c |  |
| Egg incubation period (days) *^b^* |  | 38 ± 1.5 (36-41) |  |  | 38.3 ± 1.4 (36-41) |  | 38.6 ± 1.1 (37-40) | 38.3 ± 1.0 (37-40) |  | 37.9 ± 1.5 (35-41) |  |
| No. females with fertile eggs (%) *^c^* |  | 35/37 (95)a |  |  | 9/15 (60)a,b |  | 6/7 (86)b | 10/12 (83.3)a,b |  | 35/44 (79)a,b |  |
| % egg hatching *^b,c^* |  | 71.2 ± 30.1 (0-99)a |  |  | 41.2 ± 41.3 (0-99)b |  | 7.5 ± 14.8 (0-40)c | 42.9 ± 32.5 (0-90)b |  | 55.0 ± 37.8 (0-99)b |  |

*^a^* GL: ticks exposed to *Rickettsia rickettsii-*inoculated guinea pigs during the larval stage; GN: ticks exposed to *R. rickettsii-*inoculated guinea pigs during the nymphal stage; GL+N: ticks exposed to *R. rickettsii-*inoculated guinea pigs during both the larval and nymphal stages; GC: uninfected control group, never exposed to infected hosts.

*^b^* Values presented as: mean ± standard deviation (range).

CEI: conversion efficiency index = egg mass weight/ engorged female weight × 100.

*^c^* Different letters in the same line mean significantly different values (*P*<0.05).

**Table S19.** Biological and reproductive parameters of engorged females of an *Amblyomma sculptum* tick colony from Chapada Gaúcha Municipality (NE-GSV), which was divided into four tick groups: GL, GN, GL+GN, and CG. In each group, engorged females were divided into two subgroups (infected by *Rickettsia ricketsii* or non-infected), as determined by testing the engorged female by PCR at the end of the oviposition period.

| **Biological parameter** | **Tick groups *^a^*** | | | | | | | | | |  |
| --- | --- | --- | --- | --- | --- | --- | --- | --- | --- | --- | --- |
|  | **GL** | |  | **GN** | |  | **GL+N** | |  | **GC** |  |
|  | **Infected** | **Non-infected** |  | **Infected** | **Non-infected** |  | **Infected** | **Non-infected** |  | **Non-infected** |  |
| No. recovered engorged females | 5 | 23 |  | 6 | 23 |  | 11 | 21 |  | 54 |  |
| Engorged female weight (mg) *^b,c^* | 896.4 ± 95.3  (792.6-994.8)a | 852.6 ± 155.7  (426.6-1140.0)a,c |  | 883.6 ± 135.0  (648.5-998.2)a | 781.1 ± 183.8  (534.3-1271.0)a,b,c |  | 675.5 ± 268.8  (397.1-1352.0)b,c | 698.0 ± 238.6  (182.8-1223.0)b |  | 718.5 ± 219.4  (156.9-1201.0)b |  |
| Feeding period (days) *^b^* | 11.8 ± 2.2 (9-14) | 10.2 ± 1.7 (7 - 14) |  | 8.5 ± 0.8 (8-10) | 9.2 ± 1.4 (8-13) |  | 11.6 ± 3.0 (8-16) | 9.9 ± 1.5 (8-14) |  | 9.8 ± 1.5 (8-14) |  |
| No. females that oviposited (%) | 5 (100) | 23 (100) |  | 6 (100) | 23 (100) |  | 11 (100) | 21 (100) |  | 54 (100) |  |
| Preoviposition period (days) *^b^* | 6.0 ± 1.9 (3-8) | 6.7 ± 1.0 (4-8) |  | 7.0 ± 0.6 (6-8) | 6.3 ± 1.0 (4-8) |  | 5.6 ± 1.9 (3-10) | 6.2 ± 1.2 (3-8) |  | 6.7 ± 1.2 (3-10) |  |
| Egg mass weight (mg) *^b,c^* | 491.9 ± 64.8  (426.1-586.8)a | 441.6 ± 103.6  (195.8-597.5)a,b |  | 500.9 ± 93.12  (343.1-601.3)a,b | 407.2 ± 108.1  (250.1-642.9)b,c |  | 313.0 ± 204.4  (2.4-746.4)c | 336.4 ± 166.5  (0.7-671.5)c |  | 365.7 ± 160.4  (31.5-693.1)c |  |
| CEI *^b,c^* | 55.0 ± 6.0  (45.8-61.6)a,b | 51.9 ± 8.8  (29.8-61.7)a,b |  | 56.5 ± 3.6  (52.7-60.9)a | 52.2 ± 6.9  (41.2-64.9)a,b |  | 44.8 ± 18.3  (0.4-60.8)a,b | 46.4 ± 15.4  (0.1-64.1)a,b |  | 49.7 ± 12.9  (5.3-63.6)b |  |
| Egg incubation period (days) *^b^* | 37.6 ± 1.5 (36-40) | 37.6 ± 1.8 (34-41) |  | 38.2 ± 1.3 (36-40) | 36.9 ± 1.7 (34-41) |  | 38.6 ± 1.3 (36-40) | 38.3 ± 0.9 (37-40) |  | 37.7 ± 1.5 (33-41) |  |
| No. females with fertile eggs (%) *^c^* | 5/5 (100)a | 22/23 (96)a |  | 5/6 (83)a | 22/23 (96)a |  | 10/11 (91)a | 20/21 (95)a |  | 47/54 (87)a |  |
| % egg hatching *^b,c^* | 93.6 ± 6.1 (85-99)a | 88.3 ± 21.4 (0-99)a |  | 52.6 ± 43.4 (0-95)a,c | 63.7 ± 32.2 (0-99)b,c |  | 57.1 ± 41.9 (0-99)b,c | 83.2 ± 27.9 (10-99)a,d |  | 64.3 ± 42.9 (0-99)b,c |  |

*^a^* GL: ticks exposed to *Rickettsia rickettsii-*inoculated guinea pigs during the larval stage; GN: ticks exposed to *R. rickettsii-*inoculated guinea pigs during the nymphal stage; GL+N: ticks exposed to *R. rickettsii-*inoculated guinea pigs during both the larval and nymphal stages; GC: uninfected control group, never exposed to infected hosts.

*^b^* Values presented as: mean ± standard deviation (range).

CEI: conversion efficiency index = egg mass weight/ engorged female weight × 100.

*^c^* Different letters in the same line mean significantly different values (*P*<0.05).

**Table S20**. Number of *Amblyomma sculptum* tick specimens that generated DNA sequences of the tick mitochondrial 16S rRNA gene and the nuclear second internal transcribed spacer (ITS2) according to tick colonies, and *Rickettsia rickettsii* infection status.

| **Tick**  **colony** | **No. ticks that generated DNA sequences** | | | | | **Total** |  |
| --- | --- | --- | --- | --- | --- | --- | --- |
|  | **16S rRNA** | |  | **ITS2** | |  | |
|  | **Infected *^a^*** | **Uninfected *^a^*** |  | **Infected *^a^*** | **Uninfected *^a^*** |  |  |
| E-ITU | 43 | 43 |  | 40 | 40 | 166 | |
| E-PIC | 4 | 17 |  | 3 | 8 | 32 | |
| E-PAM | 13 | 15 |  | 10 | 10 | 48 | |
| NE-PIS | 4 | 15 |  | 4 | 12 | 35 | |
| NE-POC | 9 | 26 |  | 6 | 20 | 61 | |
| NE-GSV | 25 | 22 |  | 14 | 22 | 83 | |
| Total | 98 | 138 |  | 77 | 112 | 425 | |

*^a^* infection status was determined by real-time PCR targeting rickettsial DNA in individual ticks.

**Table S21**. Absolute divergence matrix (%) between mitochondrial 16S rRNA partial sequences (410-bp) of ticks from the six tick colonies of *Amblyomma sculptum* of the present study.

| Haplotypes | NE-GSV | E-ITU | E-PAM (A) | E-PAM (B) | NE-POC | E-PIC | NE-PIS | *A. tonelliae* ^a^ |
| --- | --- | --- | --- | --- | --- | --- | --- | --- |
|  |  |  |  |  |  |  |  |  |
| NE-GSV | - |  |  |  |  |  |  |  |
| E-ITU | 2.8 | - |  |  |  |  |  |  |
| E-PAM (A) | 2.8 | 0 | - |  |  |  |  |  |
| E-PAM (B) | 0.3 | 3 | 3 | - |  |  |  |  |
| NE-POC | 0 | 2.8 | 2.8 | 0.3 | - |  |  |  |
| E-PIC | 3 | 0.3 | 0.3 | 3.3 | 3 | - |  |  |
| NE-PIS | 3 | 0.3 | 0.3 | 3.3 | 3 | 0 | - |  |
| *A. tonelliae* ^a^ | 9.3 | 10.8 | 10.8 | 9.3 | 9.3 | 10.5 | 10.5 | - |

*^a^* the 16S rRNA sequence of *Amblyomma tonelliae* (a member of the *Amblyomma cajennense* sepcies complex) from Paraguay (KF179349) was used as outgroup in the Phylogenetic tree (Fig. 2).
